# Tobamoviruses: Advances in Molecular Biology, Host Interactions and Integrated Disease Management

**DOI:** 10.3390/biology15171548

**Published:** 2026-09-04

**Authors:** Gege Li, Kunhua Zhou, Xinjie Yuan, Gang Lei, Yueqin Huang, Yu Fang, Zheng Chen, Rong Fang, Xuejun Chen

**Affiliations:** 1 Institute of Vegetables and Flowers, Jiangxi Academy of Agricultural Sciences, Nanchang 330200, China; zhoukunhua6080@163.com (K.Z.); yxinjie1115@163.com (X.Y.); leixxgang@163.com (G.L.); hyq1267@163.com (Y.H.); fyjxaas@163.com (Y.F.); chzheng2026@163.com (Z.C.); fangrong2020@163.com (R.F.); 2 Jiangxi Key Laboratory of Horticultural Crops (Fruit, Vegetable & Tea) Breeding, Jiangxi Academy of Agricultural Sciences, Nanchang 330200, China

**Keywords:** tobamovirus, TMV, ToBRFV, virus–plant interaction, genome editing, integrated disease management

## Abstract

Tobamoviruses (viruses in the genus *Tobamovirus*, family *Virgaviridae*) lead to major yield losses in economically important crops around the world. In this review, we go beyond the canonical gene expression framework by integrating recent discoveries of reverse open reading frames (rORFs) on the negative-strand RNA. These rORFs have only been experimentally validated in cucumber green mottle mosaic virus (CGMMV), with predicted sequence-conserved homologs across a subset of the genus, including TMV, ToBRFV, and PMMoV. However, they are not universally present in all tobamoviruses. We systematically dissect the infection cycle—from disassembly and replication to cell-to-cell and systemic movement—with an emphasis on the host factors hijacked at each stage. We synthesize current understanding of plant antiviral immunity, focusing on RNA silencing and NLR receptor-mediated resistance as two pillars of defense, along with the transcription factors and microRNAs that orchestrate these responses. We critically evaluate the experimental evidence for both plant defenses and viral counter-strategies, noting that many mechanistic models derive from limited model systems. We further characterize host genetic resistance and susceptibility factors applicable to crop breeding. These resources include dominant NLR and non-NLR resistance, as well as recessive resistance derived from modified host susceptibility genes. We address how viral mutations, recombination and fitness trade-offs undermine resistance durability. We then evaluate their practical deployment through conventional breeding, the exploitation of quantitative resistance, and genome editing, and outline associated agronomic drawbacks and regulatory constraints. Using ToBRFV as a case study, we analyze its epidemiological traits and assess the current arsenal of surveillance tools, from field diagnostics to remote sensing. Finally, we survey management strategies across a spectrum of maturity. Some approaches, including sanitation protocols and conventionally bred resistant cultivars, have proven effective under field conditions. The first dsRNA-based biopesticide has recently been registered in China, while other biological control agents and low-risk chemical approaches remain largely at the experimental stage. We also discuss the bottlenecks that impede lab-to-field transition and highlight promising solutions such as precision breeding and evolution-oriented cultivar deployment. By bridging molecular virology, epidemiology, and integrated disease management, this review provides a critical, bench-to-field framework for the sustainable control of tobamoviruses.

## Simple Summary

The genus *Tobamovirus* comprises many highly damaging plant viruses, including tobacco mosaic virus (TMV) and tomato brown rugose fruit virus (ToBRFV). These viruses infect diverse economically important crops, causing severe yield losses and substantial reductions in the commercial value of farm produce. In this review, we first dissect tobamovirus genome biology. We then detail the infection cycle and the host factors hijacked at each step. We also summarize recent findings regarding the ongoing “arms race” between viruses and plants: plants have developed multi-layered defense systems, and viruses have evolved counter-strategies to overcome these defenses. We further examine the genetic resistance and susceptibility factors, address how resistance-breaking variants threaten their durability, and synthesize their application through conventional breeding and genome editing. We also highlight the epidemiology of the emerging ToBRFV as a case study, along with an overview of detection and surveillance tools for tobamoviruses. Finally, we discuss control strategies at various stages of development and address both the translational bottlenecks and some solutions. By combining basic research with practical applications, this review provides valuable insights for developing sustainable management strategies for tobamoviruses.

## 1. Introduction

Plants are constantly attacked by various pests and pathogens throughout their growth cycles, and viral diseases remain one of the most challenging agricultural problems worldwide. Among these viruses, tobamoviruses stand out as both intensively studied and economically damaging, and have long served as model pathogens in plant virology. According to the International Committee on Taxonomy of Viruses Master Species List Release 41 (2025–2026), the genus *Tobamovirus* contains 37 formally recognized viral species [1]. TMV ranks first in the “Top 10 Plant Viruses in Molecular Plant Pathology”, reflecting both its long history as a research model and its continued economic impact [2]. Besides the classic TMV, the genus contains multiple destructive viruses: tomato mosaic virus (ToMV), CGMMV, pepper mild mottle virus (PMMoV) and the emerging ToBRFV. Tobamoviruses can infect numerous staple crops in the Solanaceae, Cucurbitaceae and other families, commonly triggering mosaic, mottling and necrotic lesions and resulting in obvious declines in both yield and product quality [3]. In recent years, the transboundary spread of ToBRFV has severely impacted the production of high-value crops like tomatoes, highlighting the ongoing challenge presented by tobamoviruses [4,5].

Owing to their research value and economic impact, tobamoviruses continue to attract considerable research attention in plant virology. With the development of high-throughput sequencing, researchers have successfully identified novel tobamoviruses and numerous symptomless wild intermediate hosts, helping to refine earlier interpretations about the true host range and transmission patterns of tobamoviruses [6,7,8,9]. At the same time, CRISPR-Cas-based genome editing allows researchers to probe virus–host protein interactions in a targeted way, greatly expanding our understanding of viral replication and movement [10,11,12]. These technical innovations enrich our understanding of viral pathogenic mechanisms and provide new insights for disease management.

Reflecting this growing body of work, numerous reviews have been published in recent years, yet these tend to be compartmentalized by specific subtopics. Several excellent reviews have focused on the arms race between tobamoviruses and host immunity [13,14], while others have detailed the roles of movement proteins (MPs) and coat proteins (CPs) in systemic infection [15,16]. From an applied perspective, advanced molecular diagnostics [17] and engineered resistance [18] have been thoroughly reviewed, with particular emphasis placed on ToBRFV-specific management and seed transmission [19]. More recently, epigenetic and epitranscriptomic regulation has emerged as a novel layer of antiviral defense [20]. A dedicated Special Issue, “Tobamoviruses: Molecular Aspects and Resistance Regulation”, commemorating 125 years of tobamovirus research, further synthesizes these themes through five review articles covering plant immunity, virion purification and nanotechnology, engineered resistance, overlooked viral genes, and viral movement [21]. Despite this wealth of specialized literature, a unified framework integrating evolutionary dynamics, molecular mechanisms, and field-level management strategies for the whole genus is conspicuously lacking. This review fills the critical research gap by establishing a unified bench-to-field conceptual framework that progresses sequentially from basic viral and host molecular mechanisms to translational field application. Its ten-section structure progresses sequentially from fundamental viral and host molecular mechanisms toward translational field application. Sections cover viral genome diversity, infection biology, host immunity, genetic resistance, epidemiology, integrated disease management, translational analysis, and future research priorities.

We primarily synthesize peer-reviewed literature published 2015–2026, citing some landmark early studies for foundational background. This review covers the genus broadly, incorporating findings from major tobamoviruses of agricultural significance across molecular, epidemiological, and management sections. To maintain analytical depth while ensuring balanced coverage, we anchor the review on TMV and ToBRFV. These two viruses have been most intensively studied and together provide the most comprehensive body of mechanistic, epidemiological, and resistance-related data. TMV serves as the principal reference for understanding core infection mechanisms, given its unparalleled characterization across all stages of the infection cycle. ToBRFV, in turn, offers the most extensive evidence base for resistance-breaking epidemiology and emerging disease dynamics. Other agriculturally important viruses—including ToMV, CGMMV, and PMMoV—are discussed in their respective contexts throughout the text. To avoid overgeneralization from these intensively studied models, we classify the evidence into three tiers: observations demonstrated only in a single virus; findings validated across multiple species; and mechanisms genuinely conserved across the genus. Virus-specific conclusions are flagged accordingly.

By merging virological mechanisms, epidemic dynamics and farm-level management practices, this review is intended to serve dual audiences: fundamental plant virologists and agricultural practitioners. We also aim to inspire interdisciplinary work spanning plant pathology, breeding, chemical biology and agronomy, to accelerate the conversion of lab discoveries into sustainable, long-term tobamovirus control solutions.

## 2. Diversity, Genome Organisation and Emerging Coding Capacity

### 2.1. General Genomic Architecture and Canonical Viral Proteins

Tobamovirus genomic RNA has a methylated cap structure (m^7^GpppG) at its 5′ end and forms a tRNA-like structure (TLS) at the 3′ end. These two structural features enhance RNA stability and help ensure efficient translation [22,23]. TMV, the model tobamovirus, encodes at least four proteins, organized from 5′ to 3′ as follows: the 126 kDa protein, the 183 kDa protein, the MP, and the CP. The 126 kDa protein and 183 kDa protein (collectively referred to as viral replicases) are directly translated from the genomic RNA, while the MP and CP are produced from separate subgenomic RNAs (sgRNAs) [24]. The 126 kDa protein contains a methyltransferase (Met) domain and a helicase (Hel) domain. The 183 kDa protein is produced through a read-through of an amber stop codon at the end of the 126 kDa protein open reading frame (ORF) and contains the RNA-dependent RNA polymerase (RdRp) domain [22,24]. Beyond its role in replication, the 126 kDa protein also suppresses RNA silencing by binding to silencing-associated small RNAs, thereby blocking this antiviral defense in host plants [25]. MP remodels plasmodesmata (PD) to increase the size exclusion limit from 700–800 Da to ~10 kDa to facilitate cell-to-cell viral movement [26]. As the only structural protein, CP self-assembles into left-handed helical capsids (16⅓ subunits per helical turn) to encapsidate the genomic RNA [27].

Although TMV serves as the archetypal model for tobamovirus research, comparative genomic analyses across the genus reveal substantial diversity in coding strategies, translational regulation, and the phylogenetic distribution of accessory ORFs. This diversity is particularly pronounced in the understudied minor ORFs and the recently discovered reverse-oriented ORFs, as elaborated below [6,24,28].

### 2.2. Understudied Minor Forward Open Reading Frames

The four canonical tobamovirus proteins central to most research are the 126 kDa protein and 183 kDa protein (translated from genomic RNA) and the MP and CP (expressed from subgenomic RNAs). In contrast, two long-neglected ORFs first characterized decades ago remain under-investigated; these encode the 54 kDa protein and the 4–6 kDa proteins, respectively. Their occurrence varies considerably at both the genomic and expression levels. All tobamoviruses carry the sequence encoding the 54 kDa protein. However, this discrete polypeptide has not been recovered from infected plant tissues and is detectable only via in vitro translation [28,29]. Notably, the I_1_ subgenomic RNA enabling its independent translation has only been documented in a small subset of tobamoviruses, namely TMV and sunn-hemp mosaic virus (SHMV) [28,30,31]. The small ORF6, encoding the 4–6 kDa P6 protein, was originally identified in the tobamovirus genome and investigated using in vitro translation systems. Historically, one 4–6 kDa protein were characterized from each of TMV and ToMV, and genomic screening of other sequenced tobamoviruses further identified diverse putative P6 proteins with variable amino acid lengths [28]. Morozov et al. demonstrated that the ORF6-encoded protein could be translated in vitro from synthetic RNAs mimicking the MP subgenomic mRNA or its 5′-truncated form [32]. Subsequent studies showed that internal genes of tobamoviruses can be translated through an internal ribosome entry site (IRES) located within or upstream of the MP ORF, mediating cap-independent translation [33]. Accordingly, Palukaitis et al. speculated in their review that ORF6 might be expressed through a similar non-canonical mechanism [28].

### 2.3. Emerging Coding Capacity: Reverse-Oriented ORFs

**Figure 1:**
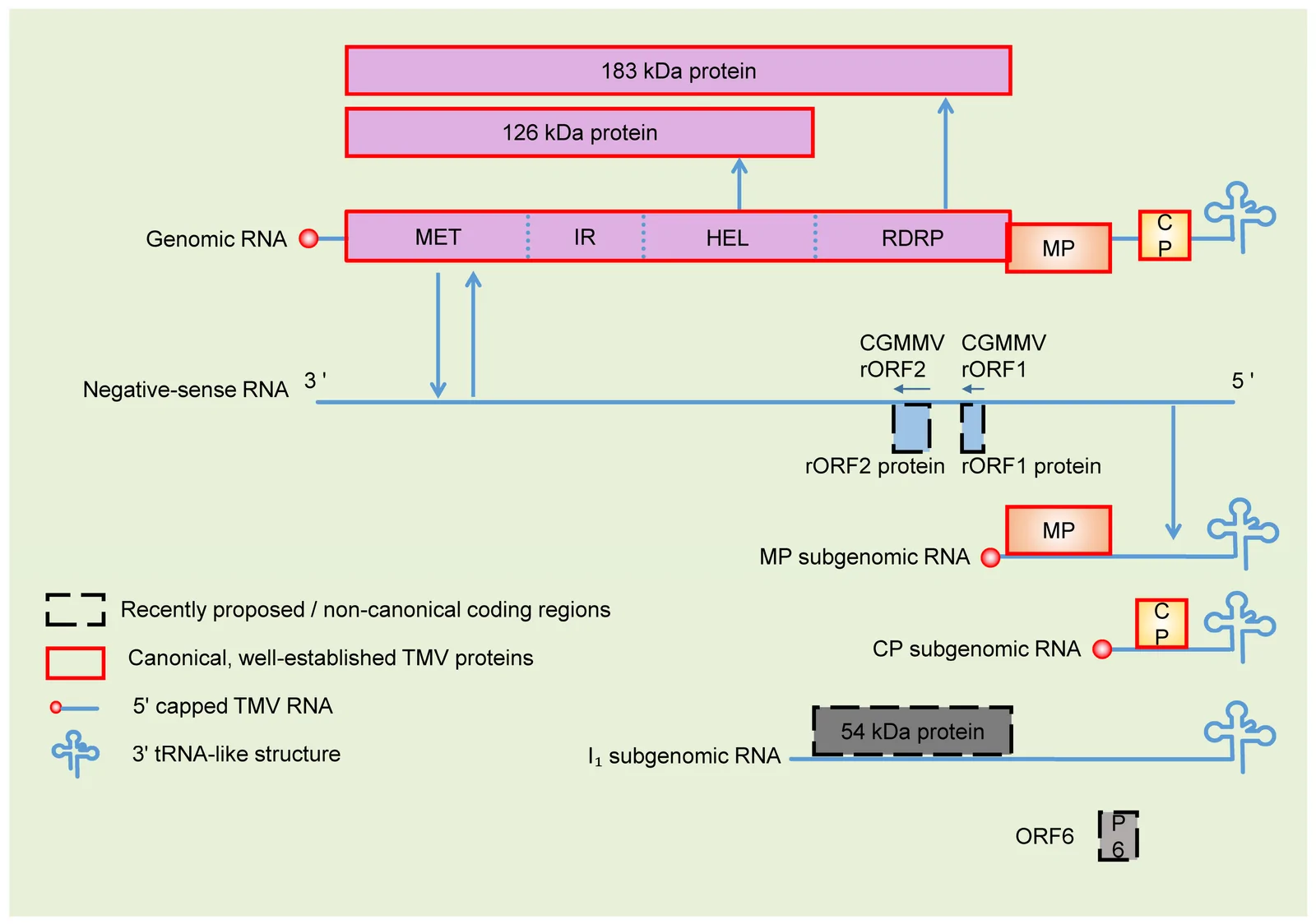
Genome organization and protein translation strategies of tobamoviruses, exemplified by tobacco mosaic virus (TMV) and cucumber green mottle mosaic virus (CGMMV). A schematic representation of the TMV genomic RNA shows the 5′ cap (m^7^GpppG) and the 3′ tRNA-like structure (TLS). The four canonical proteins are translated from distinct templates: the 126 kDa and 183 kDa proteins directly from the genomic RNA, and the movement protein (MP) and capsid protein (CP) from their respective subgenomic RNAs. The 126 kDa protein harbours a methyltransferase (MET) domain and a helicase (HEL) domain, separated by an intervening region (IR); the 183 kDa protein, arising from readthrough of the amber termination codon, carries the RNA-dependent RNA polymerase (RdRp) domain within its C-terminal extension. The 54 kDa protein (RdRp domain) is translated from the I_1_-subgenomic RNA. Although its open reading frame (ORF) is sequence-conserved across the genus, the protein is only detectable by in vitro translation, and the I_1_-subgenomic RNA has only been documented in TMV and sunn-hemp mosaic virus. The P6 protein (encoded by ORF6) lacks a dedicated subgenomic mRNA. It has been speculated that ORF6 may be translated through an internal ribosome entry site (IRES) located within or upstream of the MP ORF, mediating cap-independent translation. Its ORF shows variable presence and length across tobamoviruses, with in vitro translation demonstrated only for TMV and tomato mosaic virus. Small reverse ORFs (rORF1 and rORF2) on the negative-strand RNA of CGMMV are also shown, with functional validation currently limited to CGMMV. Blue vertical two-way arrows indicate viral RNA replication between positive-sense genomic RNA and negative-sense RNA intermediates. Up-pointing vertical arrows denote protein translation from viral positive-strand RNAs to produce corresponding proteins. Horizontal left-pointing arrows indicate the transcription direction of reverse open reading frames (rORFs) on negative-sense RNA. Down-pointing vertical arrows represent the synthesis of subgenomic RNAs.

Recent experimental research on CGMMV identified two small conserved rORFs located on the negative-strand RNA. Two independent sets of experimental data confirm the bona fide translation of rORF1 and rORF2 during CGMMV infection. These include mass spectrometry detection of unique rORF-derived peptides and ribosome profiling capturing ribosome-protected fragments mapping to negative-strand rORF loci (Figure 1). Further dicistronic reporter assays confirm that the upstream sequences of these two rORFs possess IRES activity to drive cap-independent translation. Subcellular localization assays reveal distinct subcellular distributions for proteins produced from the two rORFs. While the rORF2 protein localizes to the cell membrane and nucleolus, rORF1 colocalizes with peroxisomes, where it physically interacts with the viral 126 kD protein. This peroxisomal recruitment of rORF1 relies on the host peroxin PEX3, which facilitates the trafficking of rORF1 into peroxisomes to support viral replication [6].

Functional mutagenesis, transgenic complementation, and heterologous PVX expression assays collectively demonstrate that both rORF1 and rORF2 are required for full CGMMV virulence and systemic infection. Mutations that abolish rORF translation reduce viral accumulation and attenuate symptom severity in the natural host cucumber and the model plant *Nicotiana benthamiana*. Transgenic expression of rORF restores infectivity in rORF-deficient CGMMV mutants. Sequence alignment of 36 distinct tobamovirus species reveals that homologous rORF sequences are conserved across a defined subset of the genus, including TMV, ToBRFV, PMMoV and zucchini green mottle mosaic virus (ZGMMV), rather than being exclusive to CGMMV or universally present in all tobamoviruses. While conserved rORF coding sequences can be detected in these related species, their translation efficiency and in planta biological functions have only been fully experimentally validated with CGMMV as the model system to date. Therefore, negative-strand rORF coding capacity cannot yet be regarded as a universally established trait across the entire *Tobamovirus* genus, and broader functional verification across additional tobamovirus species remains to be performed [6].

## 3. Infection Cycle and Host Dependency

Tobamoviruses are mostly transmitted through mechanical damage [34]. Following entry through wounds or other mechanically damaged sites, virions gain access to the cytoplasm of the initially infected cells and initiate an infection cycle similar to that of other plant RNA viruses. This cycle includes several major steps: virion disassembly, translation of viral RNA, genome replication, virion assembly, cell-to-cell movement, and systemic movement to distant tissues.

### 3.1. Entry and Uncoating

The exact molecular details of how complete virion disassembly occurs in vivo remain incompletely understood. The co-translational disassembly pathway is a well-established in vitro model, derived solely from classic TMV biochemical assays; however, it has not been universally validated across all tobamovirus species. Under mildly alkaline conditions in vitro, the terminal turn of CP subunits is displaced from the virion rod, thereby exposing the 5′ leader of the RNA, which then binds the host 40S ribosomal subunit to initiate translation [35]. Subsequently, CP removal continues in the 5′-to-3′ direction, concurrent with translation of the viral replication proteins [36]. In tobacco protoplasts inoculated with purified TMV, ~70% of the viral genome is uncoated within 3 min, and progeny virions appear at about 35–40 min post-inoculation [37,38]. However, these timing data derive from a specific experimental system. They should not be generalized to all natural infections without accounting for differences in host species, tissue types, inoculation methods, and environmental conditions.

### 3.2. Translation and Replication

#### 3.2.1. Viral RNA Translation and Replication Machinery

A membrane-anchored viral replication complex (VRC) is subsequently formed through interactions among viral proteins, host endomembranes, cellular proteins, and vRNA [39,40,41]. The ultrastructure of VRCs, often referred to as viral inclusion bodies, differs among tobamoviruses. Immunocytochemical electron microscopy shows that TMV forms X-bodies, while ToMV and tobacco mild green mosaic virus (TMGMV) form distinct virus bundles [42]. Inside the VRC, negative-strand RNA is synthesized and subsequently acts as the template for generating progeny positive-strand genomic RNA and subgenomic mRNAs, which are then released into the cytoplasm [24]. Late in the replication cycle, some of the newly synthesized positive-strand RNA is packaged into viral particles [43]. Cryo-electron microscopy has revealed, at near-atomic resolution, that TMV initiates viral assembly through the AAG motif [44]. Assembly mechanisms for other tobamoviruses remain uncharacterized. Additionally, temperature modulates TMV replication efficiency. The common strain TMV-C multiplies optimally at 24 °C in *Nicotiana tabacum* leaf disks, but its RNA replication is strongly inhibited at 35 °C, where capsid protein accumulates without detectable free genomic viral RNA [45].

#### 3.2.2. Host Factors Required for VRC Assembly and Function

Several host factors are essential for VRC formation. In ToMV experimental systems, the seven-transmembrane host protein TOM1 directly interacts with the small GTP-binding protein ARL8. Consistent with biochemical evidence, TOM1 and ARL8 assemble with ToMV viral replicases into VRCs and play crucial roles in activating two core catalytic activities of the replicases: negative-strand RNA synthesis and RNA 5′ capping. Functional assays further demonstrate that viral replicases can gain these two catalytic activities only when both TOM1 and ARL8 are present [46,47,48]. TOM2A forms a conserved functional module with TOM1 to promote viral replication. Two functionally redundant TOM2A homeologs exist in tobacco, and simultaneous disruption of both paralogs drastically inhibits TMV and ToMV replication, resulting in asymptomatic plants, whereas single variants barely affect viral accumulation [11,12]. Eukaryotic elongation factor 1A (eEF1A) and eEF1B both physically associate with the shared methyltransferase (MT) domain of TMV 126/183 kDa proteins in planta. Specifically, eEF1A alone binds the conserved PK2/PK3 pseudoknot motifs within the upstream pseudoknot domain (UPD) of the TMV 3′-UTR. As a pair of coordinated host cofactors, eEF1A and eEF1B jointly facilitate the assembly of functional TMV VRCs and support efficient viral multiplication [49,50,51]. The eukaryotic translation initiation factor 3 subunit GCD10 is an indispensable component of the purified TMV RNA polymerase complex, the soluble protein core of intracellular VRC. Reciprocal co-immunoprecipitation verifies its stable binding with TMV 126/183 kDa proteins. In vitro replication assays demonstrate that anti-GCD10 antibody almost completely blocks synthesis of both positive- and negative-strand TMV RNAs [52]. The seed storage protein PAP85 functionally cooperates with the TMV 126 kDa protein to induce ER remodelling during early infection in the TMV–*Arabidopsis* system. Nevertheless, no direct physical interaction between PAP85 and the 126-kDa protein has been validated, and the universal requirement of PAP85 across diverse tobamovirus–host pathosystems remains unconfirmed [53]. VRCs require abundant membrane lipids, yet lipid trafficking from chloroplasts to ER replication sites remains poorly characterized. The chloroplast fibrillin NbLIP1 is hijacked by the CP of TMV, youcai mosaic virus (YoMV) and rehmannia mosaic virus (ReMV). Viral CP mediates NbLIP1 relocation to ER-localized VRCs, and this lipid-binding protein facilitates VRC assembly and viral accumulation [54].

### 3.3. Cell-to-Cell Movement

#### 3.3.1. MP-Mediated PD Modification and Trafficking

To establish systemic infection, tobamoviruses must cross the cell wall to infect neighboring cells. In the case of TMV, viral RNA, together with MP, viral replicases, and recruited host factors, assembles into intact VRCs at cortical microtubule-associated ER sites. From these sites, the VRCs subsequently move along the ER–actin network towards PD to facilitate cell-to-cell spread [55,56,57,58]. TMV MP interacts with the microtubule end-binding protein EB1, and overexpression of EB1 significantly inhibits TMV cell-to-cell movement [59]. For cell-to-cell movement, the intracellular trafficking of TMV ribonucleoprotein complexes is driven by class XI myosins in association with the ER network, while class VIII myosins are specifically required for MP targeting to PD [60]. However, the forms of viral complexes involved in movement may vary among different tobamoviruses and hosts. TMV MP further reduces PD-localized callose accumulation by binding host ANK protein to ease intercellular trafficking [61]. Recent evidence in the ToBRFV–tomato system shows that ToBRFV MP interacts with RabE1a and exploits RabE1a- and SEC10b-mediated exocytosis to reach the plasma membrane. There, MP engages AP2β for clathrin-dependent endocytosis toward PD. In this pathosystem, the intercellular movement unit is characterized as an MP–viral RNA complex. Whether this full pathway operates in other tobamoviruses remains to be determined, although RabE1a also interacts with MPs of TMV, ToMV and ToMMV [10].

#### 3.3.2. Host Factors Facilitating Cell-to-Cell Movement

Pectin methylesterase (PME) is a critical host factor for TMV intercellular movement. It interacts with TMV MP, and when PME expression is silenced, the spread of TMV is limited [62]. The host factors described above—EB1, ANK, RabE1a, SEC10, and AP2β—are also essential for the intracellular trafficking and intercellular transport of MP-containing complexes.

### 3.4. Systemic Movement

#### 3.4.1. Phloem Loading and Long-Distance Transport

For long-distance movement, tobamoviruses leave the mesophyll and enter the phloem sieve elements through the bundle sheath, phloem parenchyma, and companion cells. They are then passively transported with the flow of photoassimilates from source to sink tissues [63]. Both CP and MP are indispensable for long-distance movement [64,65]. Early studies showed that CP participates in systemic movement mainly by supporting virion assembly, and this structural function is critical for efficient long-distance transport [66]. Recent work suggests TMV CP may stabilize DELLA proteins to attenuate SA-triggered defenses; however, whether this mechanism operates in other tobamoviruses remains to be determined [67].

#### 3.4.2. Host Factors Required for Systemic Movement

Beyond viral proteins, many host factors play essential roles in long-distance movement. VSM1 was identified through an EMS mutagenesis screen in *Arabidopsis* as a host factor specifically required for systemic movement of turnip vein-clearing virus (TVCV) and TMV. In the recessive vsm1 mutant, TVCV and TMV replicated and moved cell-to-cell normally within inoculated leaves but were unable to enter the vasculature, resulting in a complete block of systemic infection [68]. A recessive single locus *dstm1* in the *Arabidopsis thaliana* ecotypes Columbia-0 (Col-0) restricts systemic TMV infection. In the vascular tissues of Col-0, TMV particles exhibit an abnormal curved morphology rather than the rigid rod-shaped structure of the TMV-U1 strain, indicating that DSTM1 contributes to viral particle stabilization within phloem tissues [69]. The host protein IP-L interacts with the ToMV CP to promote long-distance viral transport. Silencing of IP-L in *N. benthamiana* delayed ToMV systemic symptom development and reduced CP accumulation in systemic leaves at 7 dpi, indicating that IP-L is required for efficient long-distance movement of ToMV [70]. VIGS-mediated silencing of eEF1B in *N. benthamiana* and *Capsicum annuum* greatly reduced TMV CP accumulation and prevented systemic spread, with no infection foci detected in upper uninoculated leaves [51]. PME is also necessary for the systemic movement of TMV, specifically for viral exit from the vascular system [71]. Whether PME performs a similar role in the systemic movement of other tobamoviruses remains to be determined. A summary of the host proteins involved in various stages of the infection cycle is presented in Table 1.

## 4. Host Antiviral Immunity and Viral Counter-Defence

Upon tobamovirus infection, plants deploy a multilayered, interconnected immune system that limits viral entry, replication, and spread. This defense network can be organized into three interconnected layers: (i) basal antiviral surveillance and restriction, which includes RNA silencing, RNA decay/RNA quality control (RQC), autophagy, and dsRNA-triggered immune signaling; (ii) effector recognition and NLR (nucleotide-binding/leucine-rich repeat, NBS-LRR)-mediated immunity; (iii) regulatory integration, involving hormones, transcription factors, pathogenesis-related (PR) proteins, and miRNAs that coordinate and modulate antiviral responses. To overcome these barriers, tobamoviruses have evolved diverse counter-strategies to hinder or evade host immune surveillance.

### 4.1. Basal Antiviral Surveillance and Restriction

#### 4.1.1. Antiviral RNA Silencing and Suppression Strategies of Tobamovirus

RNA silencing is a central component of the plant antiviral immune system. During viral infection, viral double-stranded RNAs (dsRNAs) are cleaved by Dicer-like proteins (DCLs) into 21–24 nt viral small interfering RNAs (vsiRNAs). The vsiRNAs are subsequently loaded into the RNA-induced silencing complex (RISC), which contains Argonaute (AGO) proteins, and are then used as guides to specifically recognize and degrade complementary viral RNA. The host also amplifies the silencing response via cellular RNA-dependent RNA polymerases (RDRs). To achieve robust silencing, RDRs synthesize new virus-specific long dsRNAs using viral RNA templates and these dsRNAs are subsequently diced by DCLs into secondary vsiRNAs [72,73,74]. Temperature modulates the efficiency of RNA silencing by regulating vsiRNA biogenesis. Low temperature reduces the accumulation of vsiRNAs and impairs antiviral silencing, resulting in enhanced plant susceptibility to the virus, whereas elevated temperature enhances siRNA accumulation and silencing activity [75]. This amplification is supported by findings that reduced *RDR6* expression results in enhanced accumulation of TMV and turnip crinkle virus (TCV) in *Arabidopsis* [76]. *Arabidopsis RDR1* also contributes to siRNA biogenesis for TMV and cucumber mosaic virus (CMV) [77,78].

To establish a successful infection, many positive-strand RNA viruses, such as tobamoviruses, produce viral suppressors of RNA silencing (VSRs). The 126 kDa protein acts as a typical VSR by binding and capturing siRNA duplexes, which prevents the formation of the RISC and blocks RNA silencing [79,80,81]. Interestingly, TMV MP and CP can increase the expression of various RNA decay genes (including RRP41, RRP43, RRP6, DCP1, XRN3, XRN4), thereby exploiting the host’s exosome pathway to interfere with siRNA-mediated RNA silencing [82].

#### 4.1.2. RNA Decay and RQC

In plants, RNA silencing, general RNA decay, and RQC represent three major RNA degradation pathways that eliminate aberrant and foreign RNAs. Recently, general RNA decay has emerged as an independent antiviral pathway [83]. General RNA decay initiates with cytoplasmic mRNA deadenylation, followed by 3′→5′ exosomal degradation and 5′→3′ degradation carried out by decapping complexes and exoribonucleases (XRNs) [84]. Silencing of *XRN4* in *Nicotiana benthamiana* enhances local and systemic TMV infection, supporting antiviral roles of RNA decay [85].

Nonsense-mediated mRNA decay (NMD) is a well-established RQC system that monitors transcript fidelity and removes faulty RNAs, preventing the production of erroneous or toxic proteins. A recent study demonstrated that CGMMV infection activates both autophagy and the NMD pathway, and these two processes work together to suppress viral infection. Key NMD components, SMG7 and UPF3, promote viral RNA degradation by recognizing an internal termination codon and the corresponding long 3′UTR within the CGMMV genome. Intriguingly, cellular autophagy can degrade overly activated NMD factors, thereby dampening the NMD-mediated antiviral response [86]. However, these observations are currently limited to the CGMMV system; whether the same interplay operates in other tobamovirus infections remains to be determined.

#### 4.1.3. Antiviral Autophagy

Autophagy is a conserved cellular degradation process that depends on vacuoles and lysosomes to clear and recycle damaged organelles and other cellular components. Over the years, accumulating evidence has shown that autophagy also plays a key antiviral role in plants [87]. For instance, PMMoV infection can effectively activate autophagy; pharmacological or genetic inhibition of autophagy increases PMMoV accumulation and symptom severity, confirming an antiviral role [88]. In addition to limiting viral replication, autophagy regulates immune-related cell death. After TMV infection, wild-type plants exhibit a localized hypersensitive response (HR), while autophagy-deficient mutants (such as *Beclin1*, *PI3K/VPS34*, *ATG3*, and *ATG7*) develop systemic HR lesions. This evidence confirms that autophagy is indispensable for *N* gene-mediated TMV resistance by limiting the spread of host cell death [89]. In tomatoes, autophagy modulates reactive oxygen species (ROS) homeostasis and programmed cell death (PCD) during TMV infection, thereby fine-tuning ROS-associated defense responses and maintaining cellular viability [90].

A number of host regulatory proteins dynamically shape autophagy activity to balance plant antiviral immunity. Cytosolic glyceraldehyde-3-phosphate dehydrogenases (GAPCs) negatively regulate autophagy by directly interacting with ATG3, and this protein interaction can be disrupted by oxidative stress. GAPC silencing significantly strengthens N-mediated local resistance and HR against TMV, suggesting that GAPCs act as multifunctional regulators that connect autophagy, cell death and plant innate immunity [91]. Plant Bax inhibitor-1 (BI-1), a highly conserved cell death regulator, physically interacts with the core autophagy factor ATG6. Knockdown of BI-1 attenuates N-dependent TMV resistance, suppresses methyl viologen (MV)-induced autophagy, and exacerbates immune-triggered cell death. In contrast, BI-1 overexpression enhances autophagy activity but unexpectedly induces autophagy-dependent cell death. These contrasting phenotypes illustrate that BI-1 exhibits context-dependent pro-survival and pro-death dual functions [92].

The autophagy pathway acts as a key antiviral barrier during tobamovirus infection, and its modulation represents an emerging avenue for resistance breeding and disease management. Targeted modification of core autophagy-related genes (ATGs) via gene editing offers a promising strategy to enhance broad-spectrum resistance against tobamoviruses. However, given the pleiotropic roles of autophagy in plant development and stress responses, any such approach requires careful evaluation of potential agronomic trade-offs.

#### 4.1.4. Pattern-Triggered Antiviral Immune Responses

Viral dsRNA is recognized as a conserved pathogen-associated molecular pattern (PAMP) that activates pattern-triggered immunity (PTI) in plants [93,94]. Importantly, PTI triggered by dsRNA is sequence-independent, so the same immune response can be activated by viral dsRNA, in vitro-synthesized dsRNA (e.g., GFP dsRNA), and the synthetic dsRNA analog polyinosinic-polycytidylic acid [93]. The primary dsRNA receptor has not yet been identified. However, recent studies have identified multiple downstream signaling components mediating both plasmodesmal callose deposition and antiviral defense: plasma membrane-resident somatic embryogenesis receptor-like kinase 1 (SERK1), the BIK1/PBL1 kinase module, PD-located proteins 1/2/3 (PDLP1/2/3), calmodulin-like 41 (CML41) and Ca^2+^ signals. dsRNA-induced PTI restricts viral spread by triggering callose deposition at PD, thereby blocking macromolecular transport between cells [94].

Notably, dsRNA-triggered signaling differs from classical bacterial or fungal MAMP-triggered immunity in several respects. Unlike the bacterial elicitor flagellin (flg22), dsRNA does not trigger a detectable reactive oxygen species (ROS) burst, and the pathway appears to be independent of MPK3/MPK6 signaling, suggesting that different microbial patterns activate partially overlapping but distinct immune signaling frameworks [94]. Moreover, dsRNA-induced PTI operates independently of Dicer-like (DCL) proteins, distinguishing it from the RNA silencing pathway that also responds to dsRNA [93].

As a counter-defense, the MPs of three tobamoviruses—TMV, oilseed rape mosaic virus and TVCV—can suppress dsRNA-triggered callose deposition at PD, thereby facilitating infection [94].

#### 4.1.5. FLA8-Mediated Restriction of Tobamovirus Cell-to-Cell Movement

Fasciclin-like arabinogalactan proteins (FLAs) are a subgroup of arabinogalactan proteins. Genome-wide screening in *N. benthamiana* has found 38 *NbFLA* genes whose expression is markedly altered following infection by turnip mosaic virus (TuMV), implying their potential functions in biotic resistance [95]. Using a split-ubiquitin yeast two-hybrid screen, *Arabidopsis* FLA8 was identified as an interactor of turnip vein-clearing virus (TVCV) MP. FLA8 binds MP through its C-terminal GPI signal peptide (GPIsp), and the GPIsp alone is both necessary and sufficient for this interaction. Remarkably, the GPIsp can suppress MP-mediated cell-to-cell movement independently of the full-length protein. FLA8 also restricts the movement of TMV MP, indicating that it can act broadly against tobamoviruses. These findings uncover a novel antiviral mechanism in which a GPI-anchored protein, via its GPIsp domain, autonomously limits viral intercellular spread, offering new targets for engineering tobamovirus resistance [96].

### 4.2. NLR-Mediated Immunity

Plants rely on intracellular NLR immune receptors to mediate gene-for-gene specific antiviral resistance, which acts as a secondary defensive layer complementary to basal immune pathways including RNA silencing, RNA decay and autophagy. Upon direct or indirect recognition of viral avirulence (Avr) effectors, activated NLRs initiate a cascade of intracellular signalling events to trigger robust local defense responses such as reactive oxygen burst, salicylic acid accumulation and HR [97].

**Figure 2:**
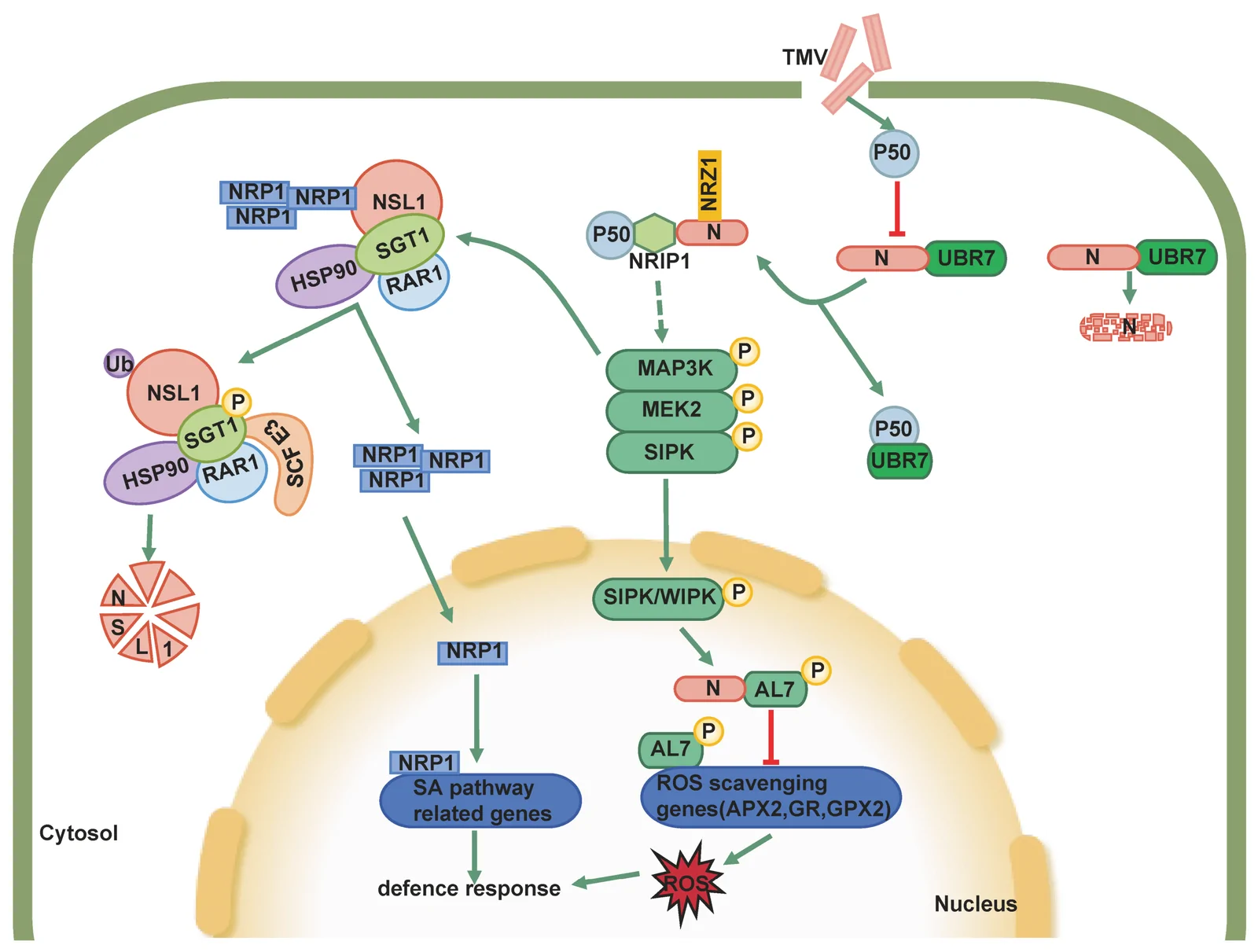
Model of N-mediated TMV resistance in tobacco. Upon TMV infection, the viral avirulence protein p50 disrupts the interaction between the N immune receptor and its negative regulator UBR7, releasing N from inhibition. The activated MAPK cascade (MAP3K-MEK2-SIPK) drives nuclear translocation of phosphorylated SIPK, which modulates AL7 activity, represses ROS-scavenging genes, and induces ROS burst and defense responses. Concurrently, the SGT1–HSP90–RAR1 complex interacts with NSL1; SIPK-mediated phosphorylation of SGT1 promotes ubiquitination/26S proteasomal degradation of the negative regulator NSL1, relieving its suppression of SA-mediated immunity via NPR1 and amplifying N-mediated resistance. The transcription factor NRZ1 further enhances N-dependent defenses. Solid-line green arrows indicate direct activation or promotion; dashed-line green arrows indicate indirect regulatory effects. Red T-bar arrows indicate inhibitory or disruptive effects on protein-protein interactions. Symbols: P, phosphorylation; Ub, ubiquitination; pie-shaped icon, targeted protein degradation; fragmented N-shaped icon, reduced abundance of the N receptor.

The dominant *N* gene confers resistance to tobamoviruses in tobacco. It encodes a Toll-like interleukin-1 receptor (TIR)-NBS-LRR immune receptor. This innate immune response is initiated by the indirect recognition of the TMV p50 helicase domain through the TIR domain of the N protein. The activity of N is negatively regulated by the E3 ubiquitin ligase UBR7; upon viral infection, the avirulence effector TMV-p50 disrupts the N-UBR7 interaction, releasing N from that negative regulation [98]. Chloroplast-localized protein NRIP1 serves as a critical host bridging factor connecting N and TMV-p50. NRIP1 is constitutively targeted to chloroplasts; however, once recruited to the cytoplasm and nucleus by TMV-p50, it physically interacts with the TIR domain of N. This interaction induces N protein dimerization and subsequently activates the downstream MAPK signaling cascade [99,100,101]. AL7 functions as a transcriptional repressor. Activated SIPK and WIPK mediate the phosphorylation of AL7 at the Ser174 residue; this post-translational modification attenuates the physical interaction between AL7 and N protein and enhances the binding affinity of AL7 for the promoters of ROS-scavenging genes. The transcriptional repression of ROS scavenging genes including *APX2*, *GR* and *GPX2* consequently triggers a ROS burst and the rapid activation of plant defense responses [102]. Simultaneously, SIPK-mediated phosphorylation of SGT1 promotes SCF-dependent ubiquitination and 26S proteasome degradation of NSL1, thereby relieving NSL1′s inhibition of SA-mediated resistance (by interfering with NPR1 nucleocytoplasmic trafficking) and further potentiating *N* gene-mediated immunity [103]. Recently characterized NRZ1, a broadly conserved transcription factor across monocots and dicots, positively regulates N-triggered defense in a way that does not require the master immune regulator Enhanced Disease Susceptibility 1 (EDS1) (Figure 2) [104].

The *N’* gene derived from *Nicotiana sylvestris* triggers a HR and confers resistance to tobamoviruses. It encodes a coiled-coil (CC)-NBS-LRR protein. The LRR domains are responsible for CP recognition, while conserved regions at the C-terminus contribute in different ways to avirulence detection [105]. CC-NBS-LRR receptors encoded by the pepper *L* genes recognize viral CP and trigger a HR, whereas tomato *Tm-2* and *Tm-2^2^* CC-NBS-LRR receptors specifically detect viral MP to activate immune signaling [106,107,108,109].

All characterized solanaceous NLR receptors exhibit prominent temperature-sensitive functional defects. Tobacco N-mediated HR is fully and reversibly abolished above 28 °C, allowing unrestricted systemic spread of TMV. Functional HR can be fully restored once temperature drops back below this threshold [110]. As the dominant commercial tobamovirus resistance gene, tomato *Tm-2^2^* loses its ability to contain TMV systemic movement at 30–31 °C in heterozygous F_1_ hybrids, and homozygous tomato varieties become fully susceptible to ToMMV at 35 °C [111,112]. Pepper *L* genes also display thermosensitive immunity. At moderate temperatures, *L^3^* and *L^4^* trigger local HR limited to inoculated leaves and confer complete ToBRFV resistance, whereas immune signalling collapses above 32 °C, leading to systemic leaf necrosis and fruit malformation [113]. The unique *L^1a^* allele shows pathotype-specific thermal stability: homozygous *L^1a^*/*L^1a^* peppers retain full resistance to P_0_-type TMV at both 24 °C and 30 °C, yet lose protection against P_1_-type PaMMV at temperatures exceeding 26 °C, a trait determined by structural disparities between viral CPs [114,115].

### 4.3. Regulatory Integration

#### 4.3.1. Transcription Factors and Hormones

Transcriptional reprogramming is a central event in plant antiviral immunity, with transcription factors (TFs) such as WRKY, MYB, and NAC acting as master switches. These TFs often function in concert with phytohormones—particularly salicylic acid (SA) and ethylene (ET)—to orchestrate a complex defense network [116]. For instance, in pepper, CaWRKYb directly activates the PR gene *CaPR10* by binding its promoter, thereby enhancing innate immunity against TMV [117]. Another pepper TF, CaBtf3, transcriptionally activates HR-associated genes (e.g., *CaAlaAT1*) and PR genes (e.g., *CaPR1* and *CaPR10*); its silencing compromises HR and increases viral accumulation [118]. Beyond serving as downstream targets of hormone signaling, some TFs also feedback-regulate hormone biosynthesis. A notable example is tobacco NAC1, which binds the promoter of isochorismate synthase 1 (ICS1), a key enzyme in SA biosynthesis, and upregulates ICS1 expression to boost TMV resistance [119]. For a broader view of additional TF family members and their hormonal links, see Table 2, which also includes the host species and regulatory evidence for each entry.

#### 4.3.2. PR Proteins

PR proteins are a class of proteins induced in plants upon pathogen infection or exposure to related stresses [130]. So far, these proteins have been classified into 17 distinct families. Their antimicrobial activity in vitro works in three main ways: acting as cell wall hydrolases, generating contact toxicity, and potentially participating in defense signal transduction [131]. Multiple PR family members are differentially induced upon tobamovirus infection across diverse plant hosts. Their induction context and intrinsic antiviral functions are fully cataloged in Table 2. Among them, a particularly well-characterized example is pepper CaPR-10, which upon TMV-P0 inoculation becomes phosphorylated and acquires RNase activity, enabling direct degradation of viral RNA and thus blocking viral proliferation [129].

#### 4.3.3. MicroRNAs

During antiviral defence responses, microRNAs (miRNAs) dynamically tune host gene expression and orchestrate multilayered regulatory circuits, ultimately steering the outcome of plant–tobamovirus interactions [132]. Importantly, infection-triggered shifts in miRNA abundance cannot be generalized to a single functional role; three distinct biological meanings underpin these expression changes.

In tobacco infected with tomato mottle mosaic virus (ToMMV), recent functional evidence has demonstrated that two endogenous miRNAs, nta-miR171c and nta-miR172a, act as host defensive regulators to limit viral proliferation. Overexpression of either miRNA significantly reduces ToMMV accumulation, whereas silencing of nta-miR171c leads to an approximately threefold increase in viral titers. Mechanistically, miR171c represses two susceptibility-associated Scarecrow-like transcription factors, NtSCL6 and NtSCL22, while miR172a targets the AP2-family protein NtRAP2-7. These two pathways contribute to basal plant immunity and thereby constrain viral spread [133].

On the contrary, many miRNAs are co-opted by tobamoviruses to weaken host resistance and create a permissive infection environment. The miR172 regulatory module exhibits striking pathosystem-specific duality. In TMV-infected tobacco, elevated miR172 suppresses the immune activator TOE3, which drives transcription of defensive genes *KL1* and *MLP43*, consequently blunting plant antiviral capacity [134]. Separately, miR6157 also negatively regulates host resistance during TMV infection: it represses the UPR component ERD2aL and impairs unfolded protein-mediated immunity, ultimately facilitating viral multiplication [135].

Beyond immune-related signaling, widespread miRNA remodelling often arises as indirect secondary consequences of infection-induced developmental and physiological stress. In TMV-challenged tissues, the MP–CP viral protein complex drives strong upregulation of miR156, miR164, miR165 and miR167, accompanied by visible stunting and mosaic symptoms [136].

## 5. Genetic Resistance and Susceptibility Factors

### 5.1. Dominant Resistance

Host dominant genetic resistance against tobamoviruses falls into two distinct functional categories: NLR-based effector-triggered immunity and non-NLR replication-inhibitory resistance. Both classes represent core genetic resources for breeding virus-resistant crops, yet their field durability is constantly threatened by viral adaptive mutations.

The tobacco *N* gene confers HR-mediated resistance to nearly all tested tobamoviruses except the TMV-Ob under standard glasshouse conditions [137]. Phylogenetic analyses reveal that tobacco *N’* gene and pepper *L* genes originate from a shared ancestral gene, with sequence divergence within LRR domains driving their differential viral recognition profiles [107]. The pepper *L* locus contains a series of dominant allelic variants following the dominance hierarchy *L^4^* > *L^3^* > *L^2^* > *L^1^*. These alleles confer resistance to distinct viral pathotypes: *L^1^* confers resistance to P_0_; *L^2^* to P_0_ and P_1_; *L^3^* to P_0_, P_1_, and P_1,2_; and *L^4^* to P_0_, P_1_, P_1,2_, and P_1,2,3_ pathotypes of tobamoviruses [138,139,140]. Resistance specificity depends on specific LRR–CP interactions, and as few as two CP mutations in a P_1,2,3,4_ pathotype PMMoV are sufficient to overcome *L^4^*-mediated resistance [141]. While *L^1^*–*L^4^* are temperature-sensitive, *L^1a^* confers stable resistance to P_0_ at both 24 °C and 30 °C [102]. The tomato *Tm-2* and *Tm-2^2^* genes, which encode CC-NBS-LRR receptors, have been widely deployed in commercial tomato cultivars. Alarmingly, the emerging ToBRFV can overcome the major *R* genes in tomato (*Tm-1*, *Tm-2*, *Tm-2^2^*) as well as those in pepper (*L^1^* and *L^2^*) [113,142,143]. However, a recent study showed that although ToBRFV overcomes *Tm-2^2^*-mediated immunity, plants carrying *Tm-2^2^* still retain partial resistance to this virus. This resistance is dose-dependent: higher *Tm-2^2^* expression leads to greater suppression of viral accumulation. This finding defines *Tm-2^2^* as a dose-sensitive, partially effective resistance gene against ToBRFV and suggests that enhancing its expression could restore more robust immunity [144].

In addition to the NLR-type immune receptors described above, plants employ non-NLR mechanisms to achieve dominant resistance against tobamoviruses. These mechanisms do not rely on immune signalling cascades but instead directly interfere with viral functions. In tomato, *Tm-1* confers resistance through a mechanism distinct from NLR-type immunity. *Tm-1* inhibits TMV accumulation in a dose-dependent manner, with homozygous plants showing stronger suppression than heterozygotes, though symptom relief does not parallel this inhibitory effect [145]. Protoplast experiments showed that two helicase-domain substitutions in the 130K/180K protein allow the escape strain TMV-Ltal to overcome Tm-1. However, the mutant replicase cannot rescue wild-type viral replication in the same cell. Thus, targeted degradation of wild-type 130K/180K protein was also excluded as the resistance mechanism [146]. Further cell-free biochemistry revealed Tm-1 directly binds wild-type 130K/180K protein and disrupts membrane replication complexes assembled with host TOM1, TOM2A and ARL8, blocking steps before negative-strand RNA synthesis [147].

Together, these examples illustrate that dominant resistance to tobamoviruses is not limited to NLR-mediated immunity but includes mechanistically diverse strategies that directly target viral replication or movement.

### 5.2. Recessive Resistance

Recessive resistance arises from loss-of-function mutations or deletion of host genes indispensable for viral propagation, thereby blocking the completion of viral life cycles. It is a common antiviral mechanism in plants [148]. Three distinct forms of recessive resistance can be distinguished based on their origin: (i) naturally occurring recessive resistance, resulting from spontaneous loss-of-function alleles in plant germplasm; (ii) experimentally induced susceptibility-gene knockdown, achieved through transient silencing (e.g., in *N. benthamiana*); (iii) genome-edited resistance, generated by targeted mutagenesis of host susceptibility genes.

#### 5.2.1. Host Factors in Viral Replication

Among the numerous host factors associated with recessive resistance, those involved in viral replication are particularly important. For instance, TOM1 and ARL8 are core components of the ToMV RNA replication complex, and both play key roles in activating the RNA synthesis and capping functions of viral replication proteins [48]. Tomato has five *TOM1* homologs. CRISPR-generated quadruple *Tom1* mutants grow normally but resist four tobamoviruses, including ToMV and ToBRFV, representing a clear case of genome-edited resistance [149]. However, this observation does not necessarily demonstrate complete agronomic neutrality across diverse field environments. Likewise, TOM1 and TOM3 in pepper are host proteins required for efficient replication, and their silencing inhibits TMV replication [150]. *TOM2A* homologs are highly conserved host factors in most plant species and facilitate TMV accumulation in host cells. Loss-of-function mutants of *TOM2A* homologs exhibit enhanced tolerance to both TMV and ToMV. This finding provides another example of genome-edited (and in some cases naturally occurring) recessive resistance [11,12].

#### 5.2.2. Host Factors in Viral Movement

Host factors involved in viral movement can also mediate recessive resistance. The *WPRb* gene—a member of the weak chloroplast movement under blue light 1 and plastid movement impaired 2-related protein family—shows a recessive association with resistance to CGMMV in watermelon. The WPRb protein localizes to PD, binds to the CGMMV MP, and facilitates viral cell-to-cell movement by regulating PD permeability [151]. This represents a case of naturally occurring recessive resistance, as it is based on genetic association in a natural host. In contrast, many other host factors have been identified through transient gene silencing. For example, eEF1A plays an important role in TMV infection and its transient silencing significantly restricts the spread of TMV [152]. CGMMV CP further manipulates host ATPδ to upregulate *NbFeSOD* and boost SOD enzymatic activity; elevated SOD eliminates excess superoxide anions and benefits viral proliferation, whereas *ATPδ* silencing impairs the systemic infection of CGMMV in *N. benthamiana* [153]. Newly identified KPILP is strongly induced after TMV and crucifer-infecting tobamovirus (crTMV) infection in *N. benthamiana*. Silencing of *KPILP* restricts viral intercellular trafficking and multiplication, and confers partial recessive resistance against tobamoviruses [154].

The major dominant and recessive resistance loci discussed above, along with their viral targets, resistance specificities, known resistance-breaking isolates, and durability status, are systematically summarized in Table 3.

### 5.3. Resistance-Breaking Mechanisms

#### 5.3.1. Molecular Basis of Resistance Breaking

Tobamoviruses evade tomato monogenic resistance through distinct viral protein mutations. Three *Tm-1* resistance-breaking TMV strains (TMV-Ltal, TMV-L_11_Y237 and TMV-CH2) contain two concurrent amino acid substitutions, p.Gln979Glu and p.His984Tyr, within the overlapping 126 kDa protein and 183 kDa protein [157]. Chimeric cDNA assays on *Tm-2^2^*-breaking ToMV-2(2) verified that the 30 kDa MP serves as the viral determinant to evade *Tm-2^2^* surveillance. Among the four divergent MP amino acids between wild-type ToMV and ToMV-2(2), only the dual C-terminal substitutions S238R and K244E are sufficient for resistance escape. The variable MP C-terminus carrying these escape sites is dispensable for viral cell-to-cell transport, demonstrating that *Tm-2^2^* relies on specific epitope recognition rather than interfering with core MP transport functions [159]. Distinct from the C-terminal escape motif of ToMV, Yan et al. (2021) demonstrated that ToBRFV MP serves as the sole virulence factor to evade *Tm-2^2^* recognition and narrowed the core resistance-breaking domain to amino acids 60–186 within its central region [161].

#### 5.3.2. Fitness Costs and Evolutionary Constraints

Viral escape mutations almost universally incur fitness costs, manifesting as impaired cell-to-cell trafficking, delayed systemic spread, or reduced replication capacity. In the ToBRFV-tomato pathosystem, the MP that enables evasion of *Tm-2^2^* recognition is inherently less active in intercellular transport than the MPs of TMV and ToMV. Hak and Spiegelman (2021) demonstrated that replacing the native MP with ToBRFV MP resulted in slower local movement and delayed systemic accumulation in susceptible tomatoes [162]. The *Tm-2^2^*-breaking determinants of ToBRFV MP reside within amino acids 1–216, a region essential for MP function including plasmodesmata targeting and RNA binding, rather than in the C-terminal domain, which is dispensable for both recognition and movement. This spatial overlap between immune evasion determinants and functional domains creates an intrinsic trade-off: ToBRFV must balance immune evasion with maintaining sufficient MP activity, incurring a measurable fitness cost even in susceptible hosts [162].

Population-level surveillance further substantiates the evolutionary constraints on resistance-breaking mutations. Fraile et al. (2011) [163] demonstrated through competition assays that the *L* gene-breaking pathotypes P_1,2_ and P_1,2,3_ exhibited substantially lower competitive fitness than the non-breaking P_0_ pathotype in susceptible pepper, with relative competitive abilities of only 0.083 and 0.002, respectively. While P_0_ and the breaking pathotypes belong to different virus species, the even lower fitness of P_1_,_2_,_3_ relative to P_1_,_2_ within the same species directly demonstrates that individual breaking mutations impose additional fitness penalties. Twenty-one years of field data revealed that P_1,2_ frequency was closely correlated with the acreage of *L^1^*-resistant cultivars: when resistant cultivars dominated, the breaking pathotype became predominant; when susceptible cultivars regained acreage, P_1,2_ abundance declined correspondingly [163]. These findings demonstrate that directional selection from large-scale resistant-cultivar cultivation can outweigh the fitness costs of breaking mutations, driving rapid fixation of resistance-breaking variants.

The presence of multiple natural weed hosts expands the viral population pool and facilitates the accumulation of rare mutations [164]. These natural reservoirs provide a larger mutational target pool, meaning that resistance-breaking variants may already be present in reservoir hosts before resistant tomato cultivars are widely deployed. Recombination during mixed infections can accelerate resistance breakdown. For example, the naturally occurring double-resistance-breaking strain ToMV1-2 carries independent *Tm-1*-breaking substitutions in the viral replicases (D1097V and R1100Q) and *Tm-2*-breaking substitutions in the MP (E52K and E133K). Chimeric virus experiments demonstrated that combining these two resistance-breaking domains from distinct single-resistance-breaking strains generates a viable double-resistance-breaking virus, confirming that recombination can accelerate resistance breakdown [156].

### 5.4. Breeding and Genome Editing

Utilizing native resistance genes to breed resistant cultivars is a sustainable and cost-effective strategy for viral disease management. At present, most breeding programs rely primarily on conventional crossing and newer biotechnological methods. This section covers three complementary strategies: traditional breeding using major resistance genes, quantitative resistance as a durable alternative, and modern biotechnologies including genome editing and transgenesis.

#### 5.4.1. Traditional Breeding

Traditional breeding has played a pivotal role in crop improvement. Researchers have spent decades identifying and mapping tobamovirus resistance genes from cultivated varieties and germplasm, with dominant resistance genes most widely applied in breeding programs. These genes have been introduced into tobacco through interspecific hybridization and backcrossing, though such materials often suffer from reduced yield and inferior quality [165]. In pepper, molecular markers linked to *L* alleles have been developed and validated for marker-assisted selection (MAS) breeding. Tightly linked SCAR markers are available for *L^3^* [166,167,168], and both SCAR and SNP markers can be used to select for *L^4^* [169,170]. In tomato, major resistance genes (*Tm-1*, *Tm-2*, and *Tm-2^2^*) have been introgressed into cultivated varieties [158]. However, viral variants quickly emerged to break these resistances. Mutant ToMV strains overcame *Tm-1*, *Tm-2* and *Tm-2^2^*, while the newly prevalent ToBRFV has defeated *Tm-2* and *Tm-2^2^* [142,143,156,160]. For this reason, screening new ToBRFV-resistant or tolerant germplasm from cultivated and wild tomato resources has become a research priority [171,172,173]. Notably, a novel dominant HREZ resistance gene derived from wild *Solanum habrochaites* has been cloned and deployed for commercial ToBRFV tomato breeding via marker-assisted backcrossing. Multiple resistant hybrids carrying this gene have been launched worldwide since 2022, yet monogenic HREZ resistance still suffers from temperature sensitivity and risks of viral mutant breakdown [174]. This constraint reinforces the demand for broader resistance resources and multi-locus resistance strategies. Gene pyramiding represents one such multi-locus strategy, which aims to stack multiple independent major resistance genes into a single cultivar. In principle, stacking distinct resistance loci raises the mutational barrier for viral escape and improves resistance durability. In practice, however, conventional gene pyramiding is labor-intensive.

In contrast to single-gene immunity, quantitative resistance is controlled by multiple QTLs. Screening of 160 tomato genotypes identified one resistant genotype (VC554) and 29 tolerant genotypes [173]. Subsequent inheritance analysis of a selected tolerant genotype (VC532) and the resistant genotype (VC554) revealed that tolerance is controlled by a single recessive gene, whereas resistance is controlled by at least two genes. An allelic test confirmed that VC532 and VC554 share a tolerance locus on chromosome 11, which explains nearly 91% of the phenotypic variation in tolerant populations but only ~41% in resistant populations, confirming the involvement of additional loci. A locus on chromosome 2, near *Tm-1*, interacts with the chromosome 11 locus to confer resistance. A GWAS in the Varitome collection identified 14 QTLs for ToBRFV resistance across eight chromosomes, with novel loci on chromosomes 1, 3, and 7 complementing previously reported regions on chromosomes 2 and 11. Five publicly available resistant accessions (BGV006370, BGV007366, LA0716, LA1777) were also discovered for breeding utilization [175]. Jaiswal et al. (2025) fine-mapped a major QTL for ToBRFV resistance in *Solanum pimpinellifolium* to a 22-kb interval on chromosome 11 at ~46.84 Mbp, with two NLR candidate genes (TIR-NBS-LRR and LRR) identified within this region [176]. The identification of these QTLs suggests that natural germplasm may harbour underutilized resistance alleles with mechanisms distinct from known *Tm* or *L* genes. Combining quantitative resistance QTLs with susceptibility-gene editing or gene pyramiding may enhance the durability of ToBRFV resistance. Nevertheless, deploying such resistance in breeding programs entails certain practical constraints, requiring extensive phenotyping, multi-environment trials, and dissection of multiple sophisticated QTLs.

#### 5.4.2. Modern Biotechnologies

Even though conventional breeding is dependable, it is also time-consuming and labor-intensive. Modern biotechnologies have significantly improved antiviral breeding against tobamoviruses in recent years. Transgenic tobacco carrying hairpin dsRNA derived from the TMV MP maintains stable full resistance up to the T4 generation under normal and low-temperature conditions [177]. CRISPR-Cas9-mediated multiplex knockout of four tomato *TOM1* homologs (*SlTOM1a–d*) confers resistance to multiple tobamoviruses, including ToBRFV, ToMV, TMV, and YoMV, in tomato [149]. As a non-transgenic alternative, an attenuated CMV-derived CR2V vector was constructed to deliver fragments of *NtTOM2A* alone or together with TMV 183 kDa protein to trigger VIGS. This approach significantly suppressed TMV multiplication without causing phytotoxicity. The inserted sequences remain stable for at least 21 days, providing a practical transient resistance option complementary to heritable genome-edited resistance [178]. Many other host proteins also assist tobamovirus infection, yet their roles are still not well understood [179]. Further study on these factors will help identify new targets for genome editing to breed virus-resistant crops.

Cross-species transfer and artificial modification expand the application potential of *R* genes. The *N* gene, originating from *Nicotiana glutinosa*, has been shown to confer resistance against TMV in transgenic tobacco and tomato, and more recently, against the emerging ToBRFV in transgenic tomato. This positions the *N* gene as a promising candidate for breeding programs aimed at developing tomato cultivars with resistance to ToBRFV [110,180,181]. Wang et al. (2025) [182] generated gain-of-function mutants of the *Tm-2^2^* resistance gene through artificial evolution, and these mutants confer resistance not only to TMV, but also to the emerging ToBRFV and its escape mutants. When combined with genome editing technologies, they are expected to speed up the development of commercial disease-resistant tomato germplasm [182]. Similarly, Li et al. (2023) [183] integrated protoplast transfection with CRISPR-Cas9 to achieve seamless knock-in editing of the tobacco endogenous *N’* gene. This workflow minimizes the risk of stable integration of exogenous plasmid backbone sequences in regenerated T_0_ plants, and the edited lines exhibit resistance to TMV-U1 [183].

Although multiplex knockout of host susceptibility genes yields robust viral resistance under controlled growth chambers, such genome-edited lines carry unresolved agronomic trade-offs that limit large-scale commercial deployment. A host gene that is dispensable under controlled experimental conditions may still contribute to plant development, stress responses, yield, or environmental adaptation under field conditions [184]. Reports that certain edited plants grow normally under specific experimental settings do not necessarily demonstrate complete agronomic neutrality across diverse environments and production systems [149]. Comprehensive field-level phenotyping across multiple seasons and environments is therefore essential before susceptibility-gene-edited varieties can be confidently deployed in commercial agriculture. Further research is needed to understand the pleiotropic effects of susceptibility gene knockouts and to identify gene family members whose loss confers resistance with minimal agronomic penalties.

Beyond technical efficacy, the practical deployment of these antiviral strategies faces divergent regulatory frameworks and public acceptance across different regions. Transgenic resistance is subject to strict GMO oversight in nearly all jurisdictions [185]. Genome-edited plants with small indels or point substitutions are regulated more leniently in several countries. By contrast, edited lines carrying exogenous sequence insertions remain under GMO supervision. The regulatory status of genome-edited crops therefore varies considerably, depending on both the modification type and national legislation [185]. Virus-induced gene silencing (VIGS) via attenuated viral vectors offers a transient, non-transgenic strategy that does not introduce heritable modifications. Consequently, it encounters fewer regulatory hurdles. However, the field deployment of attenuated viral vectors raises safety concerns, including vector stability, environmental dispersal, and potential synergy with endemic viruses [178]. Artificially evolved resistance genes, such as mutant *Tm-2^2^* alleles, can be deployed through two routes. Direct transgenesis is rapid but imposes strict GMO regulation. Alternatively, CRISPR-Cas9 can introduce the same mutations into endogenous alleles without exogenous DNA integration. The regulatory status of these approaches, however, depends on each country’s GMO definition [182]. Ultimately, region-specific risk assessment and sustained science communication are indispensable for translating these molecular innovations into sustainable tobamovirus control tools.

Besides gene knockout and knock-in, CRISPR-Cas-based genome editing has transformed antiviral breeding over the past decade. Engineered FnCas9 and its single-guide RNA (sgRNA) can target CMV and TMV, resulting in lower viral accumulation and milder disease symptoms in tobacco and *Arabidopsis* [186]. Zhang et al. (2019) [187] further designed the LshCas13a system, which cleaves TMV genomic RNA in tobacco and degrades the genomic RNAs of southern rice black-streaked dwarf virus (SRBSDV) and rice stripe mosaic virus (RSMV) in rice. This approach has successfully provided resistance to RNA viruses in both dicotyledonous and monocotyledonous plants [187].

## 6. Epidemiology, Detection and Surveillance

### 6.1. Epidemic Characteristics of ToBRFV

#### 6.1.1. Primary Inoculum

ToBRFV is an emerging tobamovirus that breaks through all known *Tm* resistance genes in tomato and *L^1^* and *L^2^* alleles in pepper. It has caused large-scale global out-breaks and poses a serious threat to solanaceous crop production worldwide [5]. The primary sources of ToBRFV include contaminated seeds and diseased plant debris. Contaminated seeds sown in previously virus-free fields can generate infected seedlings that serve as initial infection foci, while crop residues left in or on the soil after harvest retain infectious virions and provide inoculum for subsequent growing cycles [188,189].

#### 6.1.2. Viral Persistence and Reservoir Hosts

To effectively mitigate the inter-seasonal survival of ToBRFV and the formation of viral reservoirs, it is essential not only to characterize the typical phenotypes of symptomatic infections but also to pay close attention to latent infections. For susceptible tomato and pepper cultivars, typical diagnostic symptoms serve as intuitive markers to locate initial infection foci in fields. Naturally infected tomato plants develop systemic mosaic with dark green blistering and leaf narrowing or deformation. In addition, the fruits exhibit yellow or chlorotic blotches and brown rugose wrinkles or necrotic lesions [143,190]. However, symptom expression on tomato plants can vary with cultivar, plant age at infection, and environmental factors such as temperature and photoperiod [3]. Infected pepper plants exhibit stunting, leaf mottling, and puckering, while infected fruits develop irregular discoloration, necrotic blotches, and malformations. The severity of such symptoms is also regulated by temperature and plant cultivar [113,191]. Such visible disease phenotypes enable growers to rapidly trace primary inoculum sources derived from contaminated seed lots or early mechanical crop handling. In contrast, most weed hosts remain asymptomatic or exhibit only mild symptoms, making field detection of these cryptic reservoirs far more challenging.

ToBRFV exhibits strong environmental persistence that sustains these primary inoculum sources. Its infectious particles can remain active on greenhouse surfaces for up to six months and stay viable on human skin for at least two hours. The virus also survives in plant debris and soil, allowing viral reservoirs to continue releasing inoculum during fallow seasons [188]. Such high stability means that even after infected crops are removed, residual virions on structural surfaces, tools, and organic matter continue to pose infection risks for the next production cycle. This persistent trait underpins the great difficulty in eradicating ToBRFV after it invades growing facilities.

Tomato and pepper are the main cultivated crops identified as natural hosts of ToBRFV. Beyond these cultivated species, ToBRFV possesses an extremely broad experimental host range, infecting approximately 40 plant species from four families under artificial inoculation [5]. However, experimental host range data only reflect infectivity potential under controlled conditions and may not correspond to natural field infection cycles. A recent field survey in Jordan identified 12 weed species across eight families as natural ToBRFV hosts, including *Amaranthus retroflexus*, *Chenopodium murale*, *Malva parviflora*, *Solanum elaeagnifolium* and *Solanum nigrum* (*S. nigrum*) [164]. Among all infected weeds, only *S. nigrum* showed obvious leaf mosaic and mottling symptoms. Additional trials verified low seed transmission rates in *S. nigrum*, allowing this weed to sustain viral populations between cropping cycles. Field-edge weeds may act as latent primary inoculum sources. Therefore, standardized weed removal prior to tomato planting may reduce the risk of ToBRFV persisting across seasons [164]. It is worth noting that the actual epidemiological threat posed by these weed reservoirs remains unvalidated, as it hinges on whether ToBRFV harbored in various weed species can transmit to tomato crops. Recent field reports have further identified *Persicaria perfoliata* as the first validated natural host in the family Polygonaceae, and *Brassica napus* as a natural host in the family Brassicaceae, extending the known natural host range beyond the previously recognized families [192,193]. Additionally, natural infection of *Chrysanthemum indicum* has been reported in Italy, adding a new species to the Asteraceae family [194]. These findings indicate that the natural host range of ToBRFV is broader than previously recognized and may continue to expand with ongoing surveillance.

#### 6.1.3. Introduction Pathways: Seeds, Fruits and Human Activities

Contaminated seeds and vegetative plant shipments are the main pathways that bring ToBRFV into new growing areas [5,189]. Seed coat surface contamination acts as the dominant carrier driving transboundary spread. Tomato seeds harvested from symptomatic fruits may carry viral particles at a rate of 100%, yet virions predominantly adhere to the external seed coat rather than internal embryonic tissues [189]. Molecular detection assays confirm the presence of ToBRFV in seed coats and endosperm, while viral RNA is undetectable within embryos [195]. This distinction indicates that ToBRFV only causes superficial seed coat contamination instead of embryo-based vertical transmission, a critical epidemiological trait that lays a theoretical foundation for seed disinfection and cross-border phytosanitary control. Although the seedling infection rate is low in contaminated seed lots, this transmission efficiency is sufficient to initiate primary foci in newly cultivated areas [189,195].

Genomic surveillance using the ToBRFV Nextstrain build has provided direct evidence for both pathways. Seeds from Peru were identified as the most probable source of an outbreak at a tomato production site in the Netherlands, although this conclusion is limited by undersampling of this clade and the detection of the same sequence type in tomato fruits from Turkey and China [196]. Beyond seeds, fruits have also emerged as an introduction pathway. A minor phylogenetic clade revealed high homology between the virus from a UK tomato production site and sequences from imported Moroccan fruits. Since the site imports only tomato fruits and no propagation material from Morocco, this suggests that fruits may have been the source of virus introduction, although this inference requires further validation [196].

#### 6.1.4. Local Mechanical Spread

Once ToBRFV enters greenhouses or open fields, local spread mainly occurs through mechanical contact. Pruning, staking and harvesting create frequent contact risks; contaminated hands, tools, and clothing transmit the virus from infected to healthy plants through superficial wounds [197]. This transmission route is remarkably efficient: field and controlled greenhouse trials from Sicily validate that only a small number of initially infected plants can trigger full crop-wide contamination over a single growing cycle, leading to universal infection by the end of the production period [198].

Multiple phytophagous insects act as mechanical carriers without supporting viral replication inside their bodies. Pollinator buff-tailed bumblebees (*Bombus terrestris*) carry infectious virions on body surfaces and accelerate greenhouse transmission during pollination [198,199]. In addition, the polyphagous pest *Henosepilachna vigintioctopunctata* mediates cross-host ToBRFV spread. The pest retains viable virus in its digestive tract for up to 48 h, and its feeding wounds raise plant susceptibility to infection, which significantly boosts field transmission risks [200]. Notably, this insect-mediated mechanical transmission must be strictly distinguished from biological transmission, in which pathogens replicate within arthropod vectors prior to host inoculation. Neither bumblebees nor the beetle sustain internal viral proliferation. They merely transport virions externally or via regurgitation, serving only as mechanical carriers [199,200]. This classification difference delivers practical epidemiological insight: insect-borne mechanical spread is an overlooked risk source for ToBRFV outbreaks. Regular greenhouse sanitation and coordinated pest suppression can effectively reduce such cross-plot transmission risks.

#### 6.1.5. Long-Distance Dissemination: Genomic Surveillance Insights

International seed trade and cross-border transport of infected fruits and seedlings drive the long-distance global spread of ToBRFV [189,201]. This sequential chain explains the rapid global expansion of ToBRFV since its first report in Jordan in 2015, now reaching over 50 countries across four continents [170]. The phylodynamic analysis of global ToBRFV isolates in Argentina further supports this transboundary spread pattern, tracing the earliest global diversification event of the virus to June 2013 in Israel, consistent with its initial discovery in Israel and Jordan in 2014–2015 [143,202,203].

Phylogenetic analyses of geographically diverse ToBRFV isolates reveal limited global genetic variation, with all ORFs under strong purifying selection, indicating that negative selection is the dominant evolutionary force shaping the viral genome. This low nucleotide diversity supports the hypothesis that current worldwide outbreaks stem from recent, rapid transcontinental dispersal rather than long-term endemic co-evolution within isolated regional populations. Population genetic analyses further show no clear genetic differentiation among isolates from different geographic origins, with frequent gene flow between regional populations [204].

Open-source genomic surveillance platforms such as Nextstrain enable continuous tracking of emerging viral clades and provide real-time insights into transmission dynamics and evolutionary trajectories [196]. Nextstrain v4 (336 genomes, 23 countries) reveals geographic clustering, indicating multiple independent transboundary introduction routes rather than a single global wave. This pattern does not contradict the low overall genetic diversity, but rather reflects region-specific introductions via distinct agricultural pathways. The platform traced subclade 7AB to an illegal cross-protection inoculum of likely Mexican origin, and clade 7 carries resistance-breaking variants whose fitness costs may be offset by high viral loads from cross-protection, potentially accelerating their spread. However, heavy sampling bias (179/336 from the Netherlands) limits inference, underscoring the urgent need for broader global data sharing.

#### 6.1.6. Environmental Monitoring as an Early Warning Tool

Environmental monitoring has emerged as a supplementary tool to trace cryptic ToBRFV occurrence prior to official outbreak notifications. In Slovenia, high-throughput sequencing successfully detected ToBRFV RNA in agricultural wastewater before any symptomatic field cases were formally recorded, raising concerns about unrecognized cryptic viral populations and the potential for water-mediated viral dispersal [205]. This surveillance capacity is further exemplified by the recent detection of ToBRFV in municipal wastewater influent across Ontario, Canada, likely reflecting inputs from human waste and indicating widespread environmental dissemination [206].

To facilitate global genomic tracking from environmental samples, a tiled amplicon-based sequencing assay was developed, recovering 95.7% of the ToBRFV reference genome (excluding terminal 5′ and 3′ ends). This method proved robust, sensitive, and highly specific for whole-genome recovery from wastewater matrices [206]. Wastewater surveillance thus holds promise as an early warning system for incipient ToBRFV introductions. However, further empirical research is required to establish quantitative correlations between viral RNA concentrations in water samples and viable infectivity under field conditions [205,206].

#### 6.1.7. Mixed Infections and Temperature Effects

Mixed viral infections are ubiquitous under both open-field and greenhouse production conditions and substantially modify disease severity and symptom expression. Field surveys have identified natural co-infections involving ToBRFV and other viruses, including pepino mosaic virus (PepMV), tomato spotted wilt virus (TSWV), and CMV [143,207,208]. Many experiments confirm synergistic pathogenic interactions between co-infecting viruses. For example, tomato plants co-infected with ToBRFV and mild CH2 PepMV show drastically increased PepMV viral loads, which trigger severe disease signs identical to those caused by highly virulent PepMV strains. This synergistic amplification of PepMV titer was stable across all common greenhouse temperatures, and the effect was stronger when ToBRFV infected plants in advance, possibly due to the RNA silencing suppressor activity of ToBRFV proteins [209]. Similarly, simultaneous infection with ToBRFV and PaMMV accelerates symptom development of PaMMV in tomato varieties carrying the *Tm-2^2^* resistance gene [210].

Temperature is a key environmental factor that modulates the disease dynamics of tobamoviruses. Pepper plants carrying the *L^3^* and *L^4^* resistance alleles restrict ToBRFV effectively at normal temperatures through a HR. However, when temperatures reach or exceed 32 °C, this resistance is compromised. The plants then develop severe necrotic, rugose, and discoloration symptoms on stems, leaves, and fruits [113]. These observations illustrate how temperature can influence symptom expression and resistance durability, thereby promoting virus spread under high-temperature conditions. In the context of climate warming, elevated temperatures may create semi-permissive conditions that support viral replication and mutation accumulation, potentially accelerating the selection of resistance-breaking variants and further complicating epidemic management.

### 6.2. Detection and Surveillance

**Figure 3:**
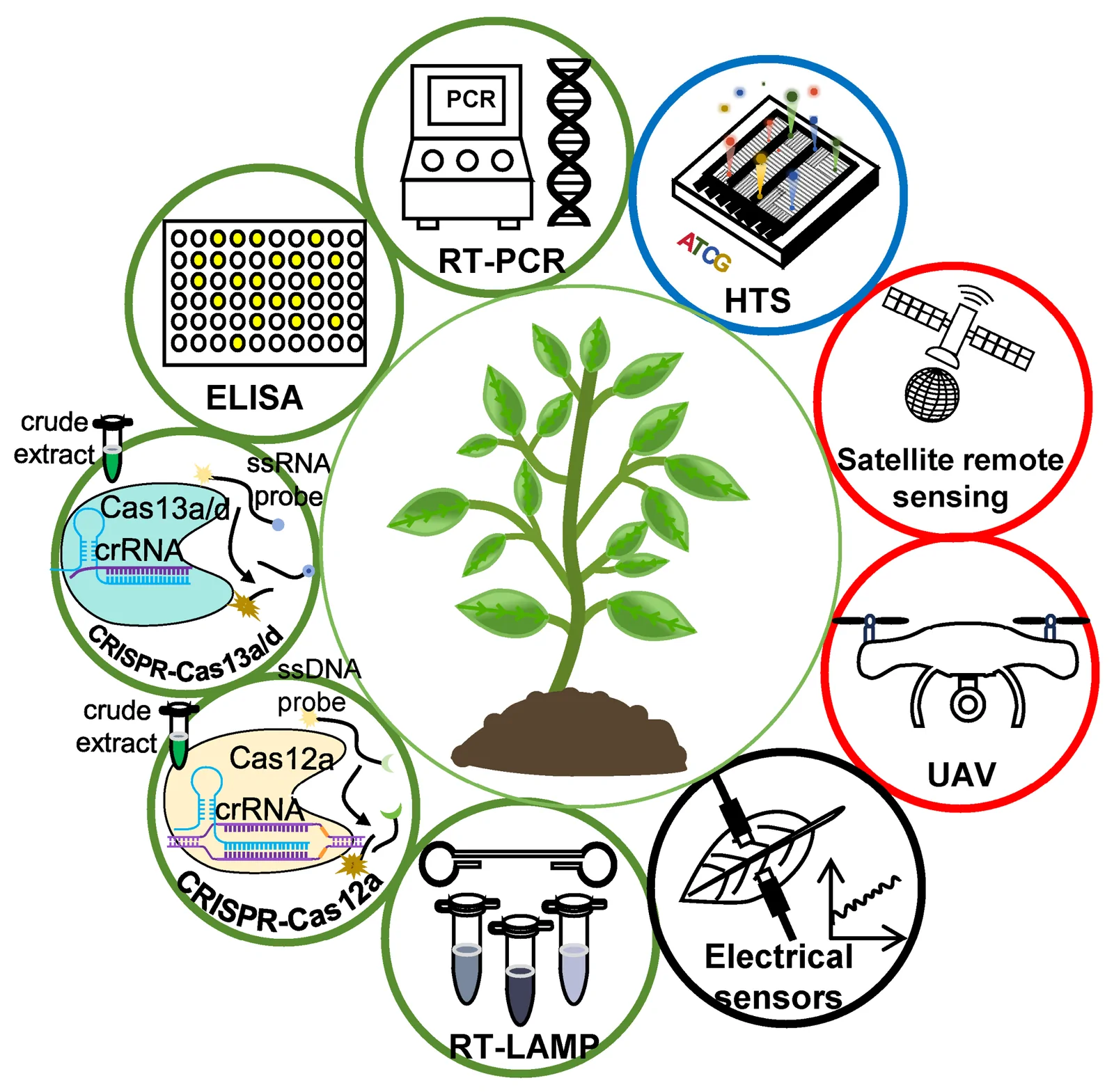
Schematic overview of detection and surveillance technologies for tobamoviruses. The four technical categories correspond to the complementary approaches described in Section 6.2.1, Section 6.2.2, Section 6.2.3 and Section 6.2.4 and are distinguished by color: green circles represent pathogen-specific diagnostics (Section 6.2.1), including ELISA, RT-PCR, RT-LAMP (reverse transcription loop-mediated isothermal amplification), CRISPR-Cas12a, and CRISPR-Cas13a/d; blue circles represent discovery-oriented surveillance (Section 6.2.2) using high-throughput sequencing (HTS); black circles represent plant-level phenotypic surveillance (Section 6.2.3) based on electrical sensors; and red circles represent landscape- and field-scale monitoring (Section 6.2.4), including UAV (unmanned aerial vehicle)-based and satellite remote sensing. Arrows in the CRISPR-Cas sub-panels depict the sequence of target nucleic-acid recognition, collateral cleavage, and fluorophore release from the cleaved reporter probe. Abbreviations: HTS, high-throughput sequencing; RT-LAMP, reverse transcription loop-mediated isothermal amplification; crRNA, CRISPR RNA; PAM, protospacer adjacent motif; UAV, unmanned aerial vehicle.

In this review, plant virus detection and surveillance technologies are categorized into four hierarchical groups: pathogen-specific diagnostics, discovery and broad-spectrum surveillance, plant-level phenotypic surveillance, and landscape-scale monitoring. Representative platforms for each group are illustrated in Figure 3.

#### 6.2.1. Pathogen-Specific Diagnostics

Pathogen-specific diagnostics detect the presence of a known virus through molecular recognition of its genetic material or proteins. These methods are highly sensitive and specific. However, they require prior knowledge of the target sequence. They are typically applied to symptomatic plants or suspected samples.

Conventional ELISA and RT-PCR require well-equipped laboratories. Loop-mediated isothermal amplification (LAMP) and recombinase polymerase amplification (RPA) support field testing. Combining these methods with CRISPR-Cas-based detection greatly enhances assay specificity. This helps to reduce false-positive results caused by non-specific amplification [211,212]. RT-LAMP is easy to use and field-deployable without specialized equipment such as thermocyclers. Visual RT-LAMP has proven effective for detecting both TMV and ToBRFV in tomato and pepper leaves and seeds [213,214]. Field-friendly detection tools also include triple immunostrips for multiple viral pathogens and integrated Penn-RAMP microfluidic chips. The fully automated chip couples RPA with LAMP and does not require manual pipetting. It reaches a detection limit of 5 copies per reaction and is ten times more sensitive than standalone LAMP, making it suitable for field screening and port quarantine [215,216]. The CRISPR-based LbCas12a, LbuCas13a and RfxCas13d assays enable TMV detection from crude plant extracts without RNA purification. While LbuCas13a and RfxCas13d allow amplification-free detection within approximately 30 min, LbCas12a detection requires prior isothermal amplification, and all systems exhibit promising potential for field-deployable diagnostics [217].

A new nanomaterial-based electrochemical immunosensor offers ultrahigh sensitivity for ToBRFV detection, with a detection limit of 1.06 fg/mL [218]. A smart decentralized diagnostic network using portable thermocyclers and RT-LAMP/RT-qPCR kits can produce lab-accurate results, supporting early warning and rapid virus containment [219].

In summary, pathogen-specific diagnostics—including ELISA, RT-PCR, LAMP, RPA, CRISPR-Cas, and nanomaterial-based biosensors—are highly sensitive and specific for known targets [213,214,215,216,217,218,219]. Field-deployable formats like RT-LAMP, RPA, and CRISPR-Cas require only minimal portable equipment (e.g., handheld heaters or visual readers). They enable rapid, on-site testing without centralized labs [213,214,215,216,217]. However, these methods have key limitations. They require prior knowledge of the target sequence. Therefore, they cannot identify unknown or emergent viruses. Nor can they fully resolve complex mixed infections, unless the virus combination has been pre-designed into a multiplexing panel. They are not designed for broad discovery. Their main value lies in validation, surveillance, and rapid response.

#### 6.2.2. Discovery and Broad-Spectrum Surveillance

Complementing targeted diagnostics, high-throughput sequencing (HTS) and metagenomics enable discovery of unknown or unexpected viruses. They provide broad-spectrum surveillance without prior assumptions about pathogen identity. These technologies are essential for identifying emerging tobamoviruses, characterizing viral diversity, and uncovering asymptomatic reservoirs.

HTS serves as a robust platform for large-scale virome profiling. It can uncover cryptic tobamovirus species and asymptomatic viral reservoirs. Wang et al. (2023) used HTS to identify two tobamoviruses, hibiscus latent Fort Pierce virus (HLFPV) and the newly reported hibiscus latent Hawaii virus (HLHV), from symptomatic *Hibiscus rosa-sinensis* [7]. Another large-scale virome survey found widespread asymptomatic tobamovirus infections including TMV, TMGMV and PMMoV across both wild and cultivated plants. Wild vegetation acts as major viral reservoirs, and habitat isolation limits cross-species viral transmission [8]. Different from traditional views, large-scale ecosystem surveys reveal that TMV, TMGMV, PMMoV and TuMV have wide host ranges, which are not connected to human activities. Seed transmission, mechanical contact, pollinators and soil survival all facilitate viral spread, offering new insights for integrated prevention [9].

However, HTS-based surveillance has both strengths and limitations. On the one hand, HTS is uniquely capable of detecting latent infections and resolving complex mixed infections. This makes it a powerful tool for early warning and virus discovery. On the other hand, sensitivity depends on sequencing depth, which may affect detection of low-titer viruses. Specificity can be compromised by reads mapping to multiple taxa or host sequences. Furthermore, while HTS can detect mixed infections, data analysis remains challenging, as complex genome mixtures may produce chimeric or incomplete assemblies.

#### 6.2.3. Plant-Level Phenotypic Surveillance

Phenotypic surveillance detects virus-induced physiological or biochemical changes at the individual plant or crop level. Electrical sensing technology can identify ToBRFV at the early latent stage, ~3 days after infection [220]. The method is simple, non-destructive, and amenable to automation. However, it detects physiological changes (impaired ion transport, altered membrane integrity) rather than the virus itself, necessitating molecular confirmation. Huang et al. (2026) used drone-based hyperspectral imaging to detect pre-symptomatic TMV in tobacco [221]. Like electrical sensing, hyperspectral imaging detects physiological anomalies rather than the virus. Such physiological anomalies frequently manifest before visible disease symptoms develop, which enables early-stage warning via phenotypic surveillance. Nevertheless, discrimination between viral infection and abiotic stresses (drought, nutrient deficiency) remains challenging, and these technologies cannot identify the specific causal virus. Instead, they serve to trigger targeted diagnostic follow-up. All positive phenotypic signals require ground-truth molecular validation.

#### 6.2.4. Landscape-Scale Monitoring

Remote sensing extends surveillance to landscape and regional scales. Unmanned aerial vehicle (UAV)-based monitoring provides high-resolution, field-level data. Drones with hyperspectral sensors can map disease distribution within individual fields [221]. However, UAV-based detection shares the same fundamental limitation as ground-level phenotypic methods: it detects physiological changes, not the virus. Satellite-based monitoring covers regional to continental scales. Using ZY-3 satellite data, researchers have generated tobacco mosaic disease distribution maps for wide-area monitoring [222].

Remote sensing detects disease-associated physiological changes rather than viruses themselves. It cannot distinguish ToBRFV from other tobamoviruses, nor can it distinguish between viral infection and abiotic stress. Environmental factors (cloud cover, soil background, illumination) substantially affect signal quality. All remote sensing-positive signals require ground-truth molecular validation before implementing field disease management strategies.

Remote sensing enables broad-area risk mapping, while in-field phenotypic surveillance provides early warning at the farm level and guides targeted sampling for confirmatory molecular diagnostics. Both are valuable for initial screening and large-scale monitoring, but they cannot replace molecular diagnostics for confirmatory identification. An effective surveillance system therefore integrates multiple layers: remote sensing for broad-area risk mapping, phenotypic sensing for within-field early detection, and molecular diagnostics for confirmatory identification.

## 7. Integrated Disease Management

**Figure 4:**
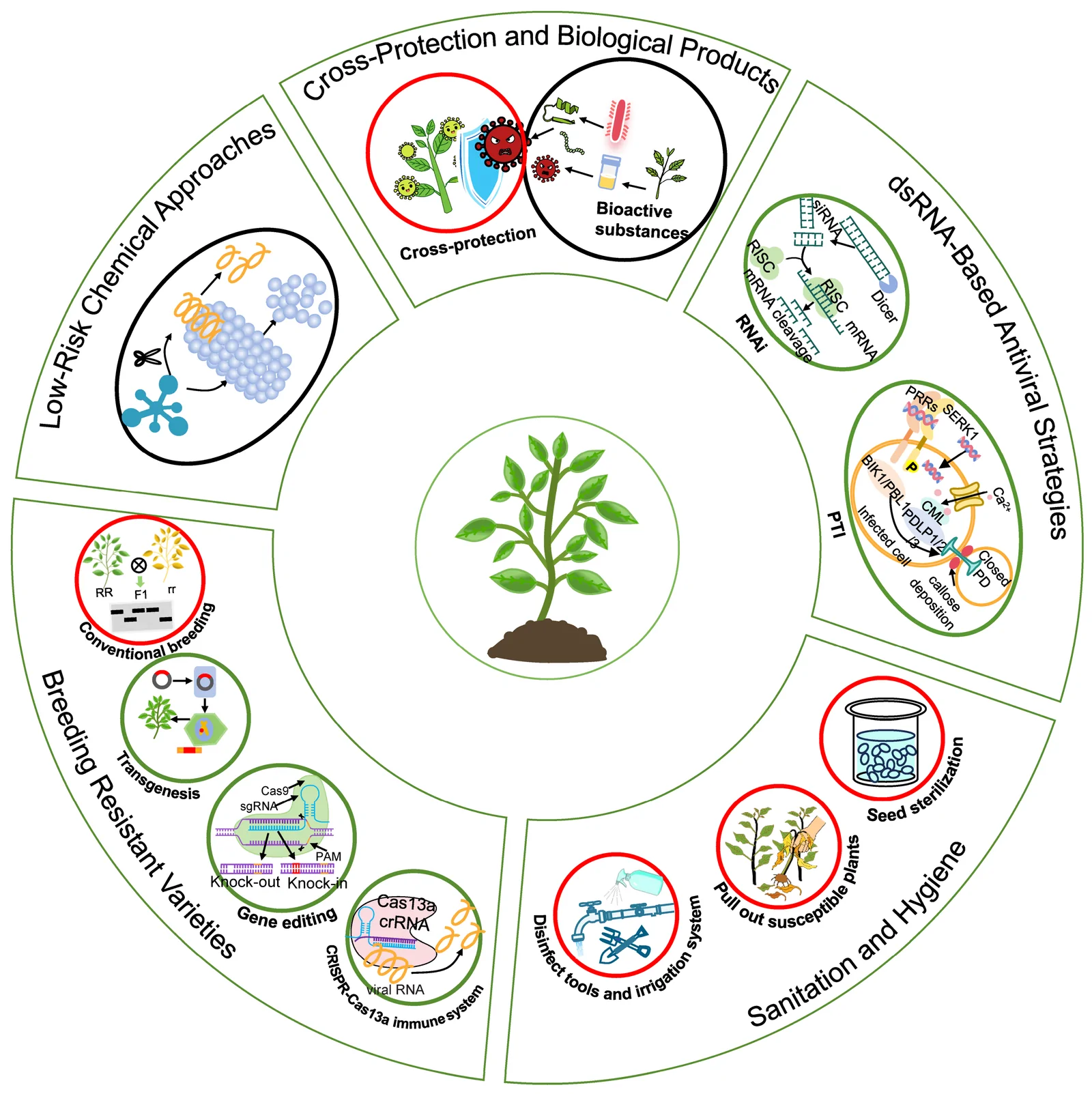
Schematic overview of management strategies for tobamoviruses. The five surrounding modules include breeding of disease-resistant varieties via conventional breeding, transgenesis and genome editing; sanitation and hygiene measures to interrupt virus transmission (seed sterilization, disinfection of tools and irrigation systems, roguing of susceptible plants); dsRNA-based antiviral strategies; cross-protection and biological products; and low-risk chemical approaches. Red circles mark strategies predominantly composed of established technologies, green circles mark strategies predominantly composed of emerging technologies, and black circles mark strategies predominantly composed of experimental technologies.

The integrated management strategies for tobamoviruses encompass a broad spectrum of approaches, ranging from exclusion and quarantine, sanitation and hygiene, deployment of resistant cultivars, RNA-based antivirals, cross-protection, and biological control products, to low-risk chemical compounds. Breeding and genetic aspects of host resistance are elaborated in chapter 5. These strategies, along with their current developmental status, are summarized in Figure 4 and further discussed in the following sections.

### 7.1. Exclusion and Quarantine

To prevent the transboundary spread of ToBRFV, several regulatory frameworks have been implemented at regional and national levels. The European and Mediterranean Plant Protection Organization (EPPO) added ToBRFV to its Alert List in January 2019 [223]. Based on a pest risk analysis, ToBRFV was subsequently included in the EPPO A2 List of pests recommended for regulation as a quarantine pest in 2020 [224]. In response to the emerging threat, the European Union adopted Commission Implementing Regulation (EU) 2020/1191 on 11 August 2020, establishing measures to prevent the introduction and internal spread of ToBRFV within the EU [225]. From 1 January 2025, however, the regulatory status of ToBRFV within the EU was reclassified from a quarantine pest to a Union-regulated non-quarantine pest (RNQP) with a zero-tolerance threshold [226]. In the United States, the U.S. Department of Agriculture Animal and Plant Health Inspection Service (APHIS) issued a Federal Order on 22 November 2019 imposing import controls on ToBRFV-contaminated plants and plant products. On 17 June 2024, APHIS amended the Federal Order (DA-2024-21), removing import requirements for tomato and pepper fruit for consumption while maintaining import requirements unchanged for propagative material [227]. International collaboration between intergovernmental bodies and national plant protection authorities aims to reduce viral introduction and transboundary spread. Improved global data sharing and standardized surveillance protocols can further mitigate cross-border transmission and reduce associated crop losses.

### 7.2. Sanitation and Hygiene

Tobamoviruses have two major transmission routes: local field spread occurs via human mechanical contact, while seed-surface viral contaminants drive long-distance cross-border pathogen dispersal [34]. Seeds collected from ToBRFV-infected fruit can carry the virus with a contamination rate of 100% under experimental conditions [228]. Mechanical transmission through contaminated soil, tools, and irrigation water can rapidly drive local outbreaks, particularly in protected cultivation [197,229]. Tobamoviruses are highly stable and may remain infectious for months or even years in plant debris and clay soil [228,230]. These interconnected and overlapping transmission pathways collectively form a complete human-mediated viral transmission chain. Therefore, standardizing farming practices, lowering viral loads in the environment, and blocking transmission pathways are all effective strategies for controlling these viruses.

Research exploring soil biochar amendments for tobamovirus control remains very limited and lacks replicated field verification. A pot-scale trial targeting ToMV documented moderate reductions in viral disease incidence and severity, while the full underlying regulatory mechanisms remain poorly characterized [231]. Seed disinfection constitutes a vital measure to eliminate primary viral inoculum. Laboratory assays demonstrated that ToBRFV-contaminated seeds treated with 10% trisodium phosphate for three hours or 2% hydrochloric acid for 30 min achieved complete elimination of surface virions, with acid treatment additionally improving seed germination and seedling vigor within trial settings [228]. However, these protocols were validated only under controlled conditions. The efficacy of these disinfection treatments may be reduced for seed lots where the virus has penetrated deeply into seed tissues, and their performance against other tobamovirus species still requires experimental verification.

Sound sanitation relies on a systematic production management framework rather than scattered disinfection operations. Whole-chain control begins with pre-planting source control, requiring growers to use certified virus-free seeds and tested propagative materials to prevent pathogen introduction. Rational zoning and segregated operational workflows are recommended to define distinct areas for clean crop production, infected-plant isolation, and waste handling. Regular replacement of disposable gloves and protective clothing during crop handling reduces the risk of mechanical virus transmission. For reusable farm implements and greenhouse surfaces, disinfection efficacy is strictly protocol-dependent. As demonstrated in previous assays targeting ToBRFV and CGMMV, effective viral inactivation requires optimized disinfectant concentrations and short contact times (≤60 s). Notably, some disinfectants effective against CGMMV performed poorly against ToBRFV, while others caused phytotoxicity. Thus, the same disinfectant cannot be assumed universally effective across all tobamoviruses or commercial settings and selection must consider virus identity, concentration, material compatibility and phytotoxicity [232]. Ozone-based irrigation water treatment can inactivate ToBRFV, yet its efficacy varies substantially with viral titer, organic load, and exposure time, and no universal protocol is adaptable to all production systems. Ozone fully eliminates infectious ToBRFV only at low virus concentrations, while high viral loads in field runoff merely experience partial suppression, leaving residual virions capable of initiating new crop infections [233]. Thus, irrigation water disinfection requires site-tailored parameter calibration, separate from surface and farm tool decontamination protocols. Once suspicious viral symptoms are observed in the field, samples should be collected for rapid molecular diagnosis to confirm tobamovirus infection. Upon positive results, targeted zoning isolation and full outbreak containment protocols must be enforced immediately to prevent large-scale secondary spread. Symptomatic seedlings and mature plants should be promptly uprooted, and crop debris and spent substrates must be sealed and transported off-site for centralized disposal to eliminate persistent viral reservoirs.

### 7.3. Resistant Cultivars

Deployment of genetically improved resistant cultivars serves as the most economical and long-term core component of integrated tobamovirus management. Conventionally bred varieties carrying *N* gene, *L* genes, or *Tm* resistance alleles can substantially reduce primary viral infection pressure in the field, while newly developed genome-edited germplasm with broad-spectrum resistance holds comparable promise for future field deployment. Nevertheless, continuous monocropping with a single resistant cultivar exerts strong selection pressure and accelerates the emergence of resistance-breaking viral isolates. Therefore, rational rotation of distinct resistant materials combined with quarantine, sanitation and RNA-based sprays is required to sustain the durability of host genetic resistance under field conditions.

### 7.4. dsRNA-Based Antiviral Strategies

dsRNA can activate both PTI and antiviral RNA silencing—two core plant antiviral defenses. Nevertheless, the induction efficiency of these two immune responses is heavily shaped by dsRNA properties, localization, cellular uptake efficiency, host species, and overall experimental context. Because of this, dsRNA-based techniques have become a promising tool for tobamovirus management. Direct delivery of virus-derived dsRNA into leaf cells via mechanical inoculation or Agrobacterium-mediated transient expression triggers antiviral RNA silencing and disrupts viral infection in a sequence-specific manner [234,235,236]. In more recent studies, bacteriophage phi6 and *Pseudomonas syringae* were engineered to produce ToBRFV-targeted dsRNA in vivo. The dsRNA produced strong antiviral activity in tomato independent of RDR6 and suppressor of gene silencing 3, and it also increased callose deposition at the PD to strengthen host defense, thereby offering a novel solution for controlling ToBRFV [237].

Naked dsRNA is highly susceptible to degradation by nucleases and UV radiation. To overcome this, non-toxic, biodegradable nanocarriers can effectively improve its stability and cellular uptake. Layered double hydroxide (LDH) clay nanosheets are a class of carrier materials with excellent performance, capable of extending the effective protection period of dsRNA to 20 days, which is far longer than the 5–7-day protection window of naked dsRNA [238]. Chitosan quaternary ammonium salt (HACC) can also be used to encapsulate dsRNA targeting TMV CP and RdRP genes. Four delivery methods, including infiltration, spraying, root soaking and pollen internalization were tested on plants. Among them, pollen internalization yielded the best control efficacy and reduced the TMV-carrying rate in seeds harvested from infected plants from 95.1% to 61.1% [239]. Single-walled carbon nanotube-based bio-carbon nanotubes serve as an alternative nanocarrier for the delivery of dsRNA. After foliar spraying, these nanocomposites move systemically through tomato tissues and keep antiviral activity in untreated upper leaves for more than 14 days, providing another practical formulation for controlling tomato viruses [240].

Despite the significant potential of exogenous dsRNA and nanocarrier-encapsulated dsRNA in tobamovirus and TMV control, this technology still faces multiple translational and industrial bottlenecks that restrict its large-scale field application and commercial promotion. First, unmodified naked dsRNA suffers from inherent environmental instability, including rapid UV degradation, nuclease hydrolysis and poor rainfastness, resulting in short field effective periods. Although LDH, HACC and carbon nanotube carriers can improve stability to a certain extent, the environmental adaptability of different formulations still needs further optimization. Second, the cellular uptake efficiency and immune activation effect of dsRNA are highly dependent on plant species, delivery methods and tissue specificity, lacking universal application protocols. Third, the strict sequence specificity of dsRNA determines its narrow antiviral spectrum, which cannot cope with mixed viral infections in fields. In addition, the high cost of large-scale dsRNA synthesis and formulation preparation is a major economic constraint for agricultural popularization. Ecologically, although existing test conditions confirm rapid biodegradation of dsRNA, potential off-target effects on non-target organisms and long-term ecological risks in complex field environments cannot be completely ruled out. Moreover, continuous application of target-specific dsRNA may drive viral sequence mutations and facilitate the emergence of resistant variants. At present, global regulatory frameworks for RNA pesticides remain incomplete, with unified safety evaluation and field application standards yet to be established, which further limits the commercial maturity and large-scale promotion of this emerging antiviral technology [241].

China’s Institute for the Control of Agrochemicals under the Ministry of Agriculture and Rural Affairs (ICAMA) has officially approved the world’s first RNA pesticide targeting TMV. It is available as a 2.5% suspension concentrate (SC) and a 65% technical concentrate (TK) [242]. The product is a dsRNA formulation targeting the TMV CP gene and is marketed under the common name Tomovircona (CAS: 3052205-09-8) [243]. According to publicly available field trial data, its inhibition rate against the target virus exceeds 85%, and the active ingredient degrades completely within 3–4 days in soil or water under tested environmental conditions [244].

### 7.5. Cross-Protection and Biological Products

#### 7.5.1. Cross-Protection

Cross-protection represents a distinct and long-established strategy for managing tobamovirus diseases. Unlike most other disease management approaches, this strategy relies on the deliberate pre-inoculation of plants with mild or attenuated viral strains to protect against subsequent infection by virulent isolates [245]. Two core mechanisms underpin cross-protection against tobamoviruses. The first is inhibition of viral disassembly mediated by the CP. The second is the host-driven RNA silencing pathway, which is closely related to VIGS. On the cellular level, attenuated strains compete for intracellular replication sites to establish superinfection exclusion (SIE), enabling stable and durable protection [246]. Xu et al. (2024) developed a stable attenuated TMV double-mutant strain (TMV-E614G-S643F) that provides effective cross-protection against wild-type TMV infection in both tobacco and tomato [247]. The attenuated CGMMV-SH33b isolate can protect melon from wild-type CGMMV [248]. In pepper, a modified mild PMMoV strain carrying several attenuating mutations produces no clear symptoms and still gives strong resistance to virulent PMMoV [249].

Despite the broad application prospects of tobamovirus cross-protection, this strategy faces multiple evolutionary, ecological and practical limitations that restrict its large-scale deployment. First, mild attenuated isolates lack sufficient genetic stability during long-term field colonization [250]. Spontaneous point mutations may revert the attenuating mutations and restore virulence. Second, when mild vaccine strains coexist with wild virulent in the host, recombination between their genomes may generate novel recombinants with enhanced fitness or altered pathogenicity, and their properties are difficult to predict in advance [251]. Third, mechanical transmission may spread the virus to adjacent non-target crops [252]. More concerningly, the host range itself could expand through mutation or recombination, enabling the attenuated virus to infect previously non-susceptible species. Fourth, cross-protection exhibits narrow strain specificity. It generally confers resistance to genetically similar viral variants yet is often ineffective against divergent isolates. Mixed infections of phylogenetically distinct viruses escape this protection and induce synergistic pathogenicity. This is a major risk for field application of cross-protection [246,252].

Genomic epidemiological surveillance has uncovered an unauthorized ToBRFV cross-protection product deployed for unregulated cross-protection practices in commercial tomato production [196]. In May 2024, the Dutch authorities imposed a penalty for the unauthorized use of such a product [253]. These findings highlight that cross-protection must be deployed within a regulated framework with case-by-case risk assessment and full compliance with phytosanitary regulations.

#### 7.5.2. Biological Products

A wide range of microbial metabolites and natural compounds have been investigated for their anti-tobamovirus activity, with most studies conducted under laboratory or greenhouse conditions; however, a few products have also been evaluated in the field. The protein Rhp-PSP from *Rhodopseudomonas palustris* JSC-3b inhibits TMV both in vivo and in vitro. This protein degrades viral CP RNA through endoribonuclease activity. Its bioactivity relies on the R129 residue and homotrimeric assembly [254,255]. Extracellular proteins secreted by *Pseudomonas fluorescens* CZ deliver distinct preventive and curative anti-TMV activity in indoor potted bioassays using *Nicotiana tabacum* cv. Samsum NN [256]. A thermostable, acid-resistant ribonuclease produced by *Bacillus cereus* ZH14 restricts TMV multiplication under laboratory test systems [257]. *Acinetobacter* strain KNF2022 and *Serratia marcescens* strain S3, which were isolated from tobacco roots, can efficiently suppress TMV infection. The secreted alkaline metalloproteinase of *S. marcescens* S3 degrades the TMV CP, disrupts viral particles and lowers viral infectivity [258,259]. Polypeptide extracts from dried mycelia of *Penicillium chrysogenum* inhibit the cell-to-cell movement of TMV by inducing callose deposition at PD [260]. Ningnanmycin (NNM) is a microbial pesticide registered in China [242]. It activates plant defense responses, including MAPK and calcium signaling. It also upregulates defense-related genes to establish systemic resistance against TMV [261].

Plant extracts also exhibit promising antiviral activity. Methanolic *Ammi visnaga* seed extract (MKSE) works directly against ToMV and strengthens host resistance in a dose-dependent way. It enhances PR-2 expression, leading to increased callose buildup and preventing viral movement between cells [262]. Isobavachalcone (IBC), a bioactive compound isolated from *Psoralea corylifolia* L., alleviates TMV-caused photosynthetic impairment in laboratory tobacco assays. When applied at the tested concentration of 40 mg/L, IBC raises chlorophyll levels and Rubisco activity while restoring multiple photosynthetic gas exchange parameters. Proteomic results verify that IBC rescues photosynthetic performance by upregulating proteins related to photosystem complexes, carbon fixation and energy metabolism [263].

Chitosan, a natural polysaccharide, acts as an effective resistance inducer against TMV. It restricts viral infectivity and CP buildup in early infection stages. It also activates endogenous proteases and RNases, producing defective viral particles with weakened infectivity [264]. Lentinan (LNT) can activate plant defenses against tobamoviruses. However, its poor leaf adhesion and low cellular uptake restrict its use in field conditions. This limitation can be addressed by combining LNT with star polycation (SPc) to form nanocomplexes. The resulting formulation inactivates TMV particles and improves plant immunity, with stable protective performance verified under open field trial environments [265]. In addition, a chitosan-coated lentinan-loaded calcium alginate hydrogel (CSL-gel) provides sustained release of LNT and Ca^2+^ over 14 days. The sustained Ca^2+^ release upregulates expression of the *calmodulin-like protein gene 3* via calcium signaling, thereby enhancing antiviral resistance. CSL-gel also promotes plant growth and confers broad-spectrum protection against TMV, tobacco rattle virus, potato virus X, and TuMV in *N. benthamiana*. All relevant bioactivity tests of this hydrogel formulation were performed only under laboratory controlled growth chamber conditions without open-field validation [266].

Most microbial metabolites, microbial strains, plant extracts, chitosan and other bioactive compounds have been tested only in laboratory or greenhouse settings. Systematic field-efficacy data, environmental fate studies, and pesticide registration information are lacking for nearly all of these agents. Two exceptions exist: ningnanmycin, a registered commercial biopesticide, and the LNT/SPc nanocomplex have both completed formal open-field trials. Conclusions drawn solely from indoor experimental data are insufficient to predict whether the remaining bioactive substances will deliver consistent control efficacy under field production conditions. The biological agents discussed above, together with the chemical compounds described in the following section, represent a broad spectrum of anti-tobamovirus strategies with markedly different maturity levels (Table 4).

### 7.6. Emerging Antiviral Compounds and Low-Risk Chemical Approaches

Low-risk chemical approaches rely on low-toxicity compounds to inactivate tobamoviruses and block their transmission in the field. Among the many low-risk chemical agents available, chlorine dioxide (ClO_2_) has been widely adopted for ToBRFV management in greenhouse and field-grown tomatoes. When sprayed onto leaves of live *Nicotiana longiflora* plants 30 min post ToBRFV inoculation, ClO_2_ at 760 mg L^−1^ produces no observable phytotoxicity. It exerts antiviral activity by oxidizing viral CPs and degrading viral genomic RNA to decrease viral replication and transmission. ClO_2_ rapidly decomposes into harmless substances in the environment. When applied to intact tomato plants, this agent lowers viral accumulation in host tissues and alleviates typical symptoms, as evidenced by the markedly reduced area under the curve of disease progress (AUCDP). It provides reliable plant protection in greenhouses and open fields by decreasing the spread of ToBRFV and mitigating crop yield losses [267].

A series of novel glaucogenin-type seco-pregnane C21 steroid derivatives developed by the Chinese Academy of Sciences exhibited potent anti-TMV activity in laboratory bioassays. Several analogues displayed superior antiviral potency to ningnanmycin in lab testing [268]. The photosensitizer chlorin e6 (Ce6) was shown to bind to a G-quadruplex (G4)-forming sequence in the TMV genome. Under LED-light irradiation, Ce6 generates reactive oxygen species (ROS) that selectively cleave the G4-containing RNA, resulting in reduced viral accumulation and alleviated viral symptoms in tobacco plants cultivated in greenhouses [269]. Synthetic cytidine peptide derivatives were screened for anti-TMV activity in *Nicotiana glutinosa*. Among them, derivative SN11 exhibited marked protective, inactivating, and curative effects, demonstrating its ability to act at multiple stages of the virus infection cycle. Furthermore, SN11 inhibited the systemic infection of TMV in *Nicotiana tabacum* plants [270]. Several synthetic ferulic acid dimers exhibited anti-TMV activity in laboratory biological tests. Compound A6 exerted the most prominent viral inhibitory effect among the tested analogues. Lab-based interaction assays verified that A6 tightly binds TMV CP to disrupt viral particle integrity in vitro [271]. For the newly synthesized compounds mentioned above (seco-pregnane derivatives, Ce6, SN11 and ferulic acid dimers), comprehensive evaluations are still lacking. These include field efficacy, environmental fate, toxicology, formulation and pesticide registration. Until such comprehensive validation data are available, their practical agricultural applicability cannot be reliably extrapolated from laboratory-only results.

All biological control agents and synthetic low-risk chemical antiviral strategies covered in Section 7.5 and Section 7.6 are collated in Table 4 for cross-comparison of their mechanisms, validation grades, practical advantages, constraints and commercial registration status.

## 8. Translation from Proof of Concept to Field Implementation

Numerous antiviral strategies against tobamoviruses exhibit robust inhibitory effects under strictly controlled laboratory and greenhouse environments. Translating these laboratory discoveries into practical, scalable, and cost-effective field solutions remains a major challenge.

### 8.1. Core Discrepancies Between Laboratory and Field

The gap between controlled conditions and complex production environments is a major translational barrier. In the laboratory, the temperature, humidity, light levels and pathogen pressure are all strictly controlled. The plants are usually homozygous inbred lines, and inoculations are performed under strictly controlled conditions with defined inoculum concentrations. Field conditions, however, are markedly different. Field crops undergo daily and seasonal shifts in temperature and humidity. They grow in heterogeneous soils and compete with surrounding weeds. Moreover, mixed infections by diverse pathogens commonly occur under field conditions. Commercial hybrid cultivars have heterozygous genetic backgrounds. As a result, the same control measure often shows variable efficacy across different varieties—a variability that is rarely captured in single-genotype laboratory tests. Residual virions on plastic mulch, trellis cords, reusable growing trays, and irrigation pipelines act as persistent inoculum sources. Mechanical transmission via contaminated tools, irrigation water, and infected debris may further sustain elevated inoculum pressure throughout the growing season. Pollinators including bumblebees and honeybees may carry infectious virions on their body surfaces and mediate mechanical transmission between plants. This process can accelerate within-field viral spread and reduce the performance of spray-based management measures.

### 8.2. Translational Bottlenecks for Major Technologies

The chemical synthesis of dsRNA remains costly, and the extraction processes for bio-derived compounds are difficult to standardize. The industrial-scale production of nanocomposite formulations is also too expensive for smallholder farmers. Similar issues apply to most biological control agents and natural products. With the exception of ningnanmycin and the lentinan-polycation complex, almost all of these substances lack systematic field efficacy data and environmental fate studies [261,266]. Tomovircona is registered for use in China; however, its strict sequence specificity restricts activity exclusively to TMV. Mixed infections, which are common in the field, are not covered by this product. Attenuated tobamovirus strains only confer protection against homologous genotypes. Under mixed infections, they fail to suppress divergent field isolates, and co-infection often exacerbates disease severity [252]. Furthermore, in the field, attenuated strains may undergo reversion mutations, recombine with wild-type viruses, or spread to neighboring crops through routine agricultural practices. Additionally, most precision technologies demand specialized training, and limited outreach services raise labor requirements and lower farmer adoption rates. Regulatory frameworks vary across technologies and regions. Transgenic crops are strictly regulated as GMOs in nearly all countries [110,177,180]. Genome-edited plants with large exogenous insertions remain under full GMO oversight [183]. There are currently no globally harmonized risk assessment standards for attenuated live-virus vaccines or foliar dsRNA pesticides. Most crops lack standardized procedures for the propagation of attenuated strains and the uniform inoculation of seedlings.

### 8.3. Resistance Durability and Virus Evolutionary Risks

Biological distinctions among immunity, partial resistance, quantitative resistance, and tolerance carry important implications for crop breeding. Tolerant genotypes reduce visible symptoms yet cannot effectively block viral replication. Nevertheless, compared with resistance, tolerance may reduce the selection pressure on virulent isolates and could provide a broader and more stable phenotype [272,273]. Quantitative resistance is controlled by multiple genes and imposes relatively weak selective pressure on viral populations, which slows the emergence of resistance-breaking variants and extends field durability under commercial cultivation. In contrast, individual *R* genes (including naturally occurring alleles and engineered immune receptors) usually confer nearly complete immunity in greenhouse trials. Nevertheless, they exert intense directional selection on viruses, rendering cultivars highly vulnerable to resistance breakdown under large-scale monocropping systems [274]. Mixed field infections may further facilitate viral recombination, driving the rapid evolution of variants capable of evading single *R*-gene recognition and drastically shortening the commercial lifespan of resistant cultivars. Recessive resistance, derived from loss or modification of host susceptibility factors, is generally more durable, as the virus cannot easily compensate for the missing or altered host function. A recent study on ToBRFV demonstrated that a single recessive locus conferring tolerance could be combined with the *Tm-1* locus on chromosome 2 to achieve complete resistance. Plants carrying the tolerance locus alone remained symptomless but sustained high viral titers, whereas the combination of the tolerance locus with *Tm-1* reduced viral accumulation to near-undetectable levels. The authors further highlighted that the participation of tolerance in the resistance phenotype may reduce the selection pressure for virulent isolates and enhance phenotypic stability [173]. Collectively, these results indicate that future breeding strategies may benefit from stacking tolerance loci, quantitative trait loci (QTLs), and major *R* genes to extend the field longevity of resistant cultivars in commercial production.

Multiple breeding strategies have been proposed to address resistance breakdown caused by viral evolution, including the mining of novel resistance genes, gene pyramiding, susceptibility gene knockout, and engineered immune receptors. Among these approaches, deployment of novel *R* genes represents a well-established strategy that combines conventional breeding with marker-assisted selection (MAS), and corresponding commercial cultivars are already available [174]. Gene pyramiding holds theoretical promise to raise the mutational barrier for viral escape, yet its practical implementation remains labor-intensive within conventional breeding frameworks. By contrast, susceptibility gene editing and engineered immune receptor technologies remain largely confined to laboratory research. Although these strategies can partially reduce viral evolutionary pressure, the long-term performance of the resulting germplasm under complex field conditions with co-occurring viral species remains incompletely validated.

In summary, continuous monocropping without coordinated phytosanitary measures may accelerate adaptive viral evolution, a key constraint that impairs the long-term stable field performance of resistant germplasm. Therefore, routine surveillance of viral population dynamics is essential when deploying newly developed resistant cultivars for commercial production.

### 8.4. Integrated Strategies to Accelerate Translation

Bridging the gap between laboratory research and field production requires systematic strategies. These strategies span breeding, antiviral agents, cost control, detection, and long-term virus monitoring. Within breeding pipelines, edited susceptibility genes can be combined with pyramided resistance QTLs. To extend resistance durability, growers should adopt mixed planting or annual rotation of varieties with diverse resistance loci. For dsRNA and plant-derived antiviral agents, biodegradable nanocarriers and slow-release hydrogels can prolong field efficacy [239,266]. A range of emerging low-risk chemical approaches may also serve as promising supplementary measures for mitigating tobamovirus infections under field conditions. Low-cost bacterial fermentation can lower dsRNA manufacturing expenses [237]. Automated water circulation disinfection systems and UAV-mediated dsRNA spraying reduce human operational error and field management costs. Supportive industrial policies, including standardized seed health certification criteria, subsidised antiviral biopesticides and a system of regular technical training for farmers, are useful in bridging the gap between laboratory proof-of-concept research and practical field application. Harmonized field trial datasets are also needed to support comparative research and the regulatory registration. Collectively, these multifaceted interventions reduce field viral loads and constrain viral adaptive evolution, thereby delaying resistance breakdown. Collectively, these multifaceted interventions reduce field viral loads and constrain viral adaptive evolution, thereby delaying resistance breakdown. This evolution-aware integrated framework enables sustainable field protection and is particularly relevant for managing highly aggressive tobamoviruses such as ToBRFV.

## 9. Future Priorities

Despite substantial progress, several critical unresolved questions continue to limit mechanistic understanding and the practical disease management.

First, rORFs have well-validated protein-coding and virulence-related functions in CGMMV, and homologous nucleotide sequences can be detected in a subset of other Tobamovirus species, as outlined in Section 2.3. Thus, the priority questions are: Are these non-canonical ORFs translated and functional in other tobamoviruses, and do they contribute to virulence or host adaptation in a species-specific manner? Resolving these questions requires systematic cross-species functional validation—extending the mutagenesis, transgenic complementation, and localization assays already performed in CGMMV to other tobamoviruses that harbor homologous rORF sequences.

Second, the identification of host susceptibility factors has opened new avenues for recessive resistance breeding, yet the central question remains: Which of these factors can be edited without incurring unacceptable agronomic trade-offs? Comprehensive field-level phenotyping of edited lines across multiple environments is urgently needed to resolve this trade-off. A further central evolutionary question follows: How can broad-spectrum host resistance be designed and deployed to maintain durability under field-scale viral selection pressure? Further empirical evaluation of strategies combining quantitative resistance, susceptibility-gene editing and rational cultivar rotation will help mitigate rapid viral adaptive evolution driven by large-scale cultivation.

Third, within disease surveillance and phytosanitary management, two interconnected practical gaps persist: Can field-deployed diagnostic workflows reliably detect incipient infections prior to virus-mediated within-crop spread? And how should routine viral genomic surveillance be formally integrated into resistance-breeding pipelines and official quarantine frameworks to anticipate resistance-breaking variants and transboundary virus movement? Validated end-to-end operational pipelines connecting pre-symptomatic detection, population-scale genomic monitoring and regulatory intervention are still lacking.

Finally, for experimental antiviral approaches, the major translational bottlenecks remain: How can dsRNA formulations achieve economically feasible environmental persistence and efficient in planta delivery? More broadly, how can promising antiviral activity observed in laboratory assays be translated into consistent, reproducible efficacy across geographically diverse multi-location field trials? These challenges apply not only to dsRNA-based products but also to microbial biocontrol agents, plant-derived antiviral compounds, and low-risk chemical agents. Addressing them requires improved formulation engineering and rigorous multi-site field validation prior to large-scale agricultural deployment.

## 10. Conclusions

Tobamoviruses have long represented a core model in plant virology, and research on this genus has delivered key advances spanning fundamental research and practical applications. Recent advances have clarified the functions of canonical viral proteins, uncovered novel coding potential of the rORFs, and identified a growing repertoire of host factors hijacked at each stage of the infection cycle. In parallel, plant antiviral immunity is organized into interconnected layers, including RNA silencing, autophagy, and NLR receptor-mediated resistance. Transcription factors, PR proteins, and microRNAs coordinate these immune responses, whereas viruses have evolved counterstrategies to evade or suppress host defenses. Collectively, this detailed mechanistic understanding identifies novel molecular targets for intervention. Yet the core challenge persists: How can these discoveries be translated into durable, scalable, environmentally sound, and epidemiologically effective field management practices? Bridging this bench-to-field gap will govern whether next-generation resistance strategies, surveillance platforms, and integrated management practices provide sustained protection under field conditions. Achieving this will rely not only on continued molecular innovation but also on systematic attention to translational bottlenecks, evolutionary dynamics, and the regulatory and socioeconomic contexts that shape disease management.

## Figures and Tables

**Table 1:** Host factors involved in the infection cycle of tobamoviruses.

Host Factor	Host Species	Virus Species	Interacting Viral Protein(s)	Biological Function	Infection Stage(s)	Type of Evidence	Effect of Loss of Function	Key References
TOM1	*Arabidopsis thaliana*, *Nicotiana tabacum*	TMV-Cg, TMV-L, ToMV	126 kDa protein, 183 kDa protein	Recruits and anchors replication proteins on intracellular membranes to form replication complexes	Replication	Physical interaction; genetic evidence	Loss-of-function *Tom1* mutant strongly reduces the accumulation of TMV	[46,47,48]
ARL8	*Arabidopsis thaliana*, *Nicotiana tabacum*	ToMV	126 kDa protein, 183 kDa protein	Cooperates with TOM1 to activate the RNA capping and RdRp activities of viral replicases	Replication	Physical interaction; knockout	Double mutant (*arl8a arl8b*) and triple mutant (*arl8a arl8b arl8c*) fully abolish detectable TMV-Cg and ToMV CP accumulation in whole inoculated *Arabidopsis* plants	[48]
eEF1A	*Nicotiana tabacum*, *N. benthamiana*, *wheat germ*	TMV	126 kDa protein, 183 kDa protein	Participates in VRC assembly	Translation and Replication	Physical interaction; silencing	VIGS greatly reduces viral CP accumulation and virus spread	[49,50,51]
eEF1B	*Nicotiana benthamiana*, *Capsicum annuum*	TMV	126 kDa protein, 183 kDa protein	Participates in VRC assembly	Replication and systemic infection	Physical interaction; silencing	VIGS greatly reduces viral CP accumulation and virus spread	[51]
TOM2A	*Nicotiana tabacum*, *Solanum lycopersicum*	TMV, ToMV	Indirect (via TOM1; 126 kDa protein, 183 kDa protein)	Interacts with TOM1 to enhance virus accumulation	Replication	Knockout; genetic evidence	Single *TOM2A* mutants support wild-type viral replication and exhibit mosaic symptoms; dual homeolog knockout almost abolishes TMV accumulation and confers an asymptomatic phenotype. Tomato *SlTOM2A* knockouts show suppressed TMV/ToMV with no mosaic symptoms	[11,12]
GCD10	*Lycopersicon esculentum*	TMV	126 kDa protein, 183 kDa protein	Component of VRC	Replication	Physical interaction; biochemical inhibition	Anti-GCD10 antibodies blocked GCD10 function and inhibited dsRNA and ssRNA synthesis by >95% in a concentration-dependent manner	[52]
PAP85	*Arabidopsis thaliana*	TMV	Unknown	Functionally cooperates with TMV 126 kDa protein to induce ER transition and facilitate TMV accumulation	Replication	Silencing; functional rescue	*PAP85* knockdown (stable RNAi lines) reduced TMV accumulation	[53]
NBLIP1	*Nicotiana benthamiana*	ReMV, YoMV, TMV	CP	Supplies lipids to promote VRC formation	Replication	Physical interaction; overexpression; silencing	VIGS of *NbLIP1* significantly reduces viral replication, viral protein accumulation, and VRO formation	[54]
EB1	*Arabidopsis thaliana*, *Nicotiana benthamiana*	TMV	MP	Regulates microtubule cytoskeleton	Cell-to-cell movement	Physical interaction;	No direct loss-of-function data	[59]
ANK	*Nicotiana tabacum*	TMV	MP	Reduces callose deposition at PD	Cell-to-cell movement	Physical interaction; overexpression; silencing	RNAi suppression of *ANK* substantially reduced MP cell-to-cell movement	[61]
PME	*Nicotiana tabacum*	TMV, TVCV	MP	Facilitates viral cell-to-cell movement and promotes virus release from vascular tissues for systemic spread	Cell-to-cell movement, Systemic movement	Physical interaction; silencing	Antisense suppression of *PME* delayed TMV systemic movement	[62,71]
RabE1a	*Solanum lycopersicum*, *Nicotiana benthamiana*	ToBRFV, TMV, ToMV, ToMMV	MP	Regulates exocytosis to deliver MP to plasma membrane	Cell-to-cell movement	Physical interaction; overexpression; silencing; knockout	*RabE1a* knockout restricts ToBRFV infectivity in tomato; silencing of *RabE1a* in *N. benthamiana* impairs MP PM localization, reduces tobamovirus cell-to-cell movement, and decreases infection foci size	[10]
SEC10	*Solanum lycopersicum*, *Nicotiana benthamiana*	ToBRFV	Indirect (via RabE1a; MP)	Directly interacts with RabE1a to regulate exocytosis to deliver MP to plasma membrane	Cell-to-cell movement	Silencing	Silencing of *SEC10b* in tomato and *N. benthamiana* significantly reduces CP accumulation and cell-to-cell movement	[10]
AP2β	*Solanum lycopersicum*, *Nicotiana benthamiana*	ToBRFV	MP	Mediates clathrin-dependent endocytosis of MP from the plasma membrane to PD	Cell-to-cell movement	Physical interaction; silencing	Tomato *SlAP2β* silencing: reduced ToBRFV CP accumulation and milder symptoms; *N. benthamiana NbAP2β*-KD: ToBRFV-GFP infection foci significantly reduced	[10]
DELLA proteins	*Nicotiana benthamiana*, *Arabidopsis thaliana*	TMV	Unknown	CP-mediated suppression of SA signaling stabilizes DELLA proteins, which in turn facilitates long-distance movement	Long-distance movement	Overexpression	Direct loss-of-function data for DELLA proteins in systemic movement are not provided	[67]
VSM1	*Arabidopsis thaliana*	TVCV, TMV	Unknown	Helps virus entry into vascular tissues	Long-distance movement	Genetic evidence	The recessive *vsm1* mutation blocks systemic movement of TVCV and TMV	[68]
DSTM1	*Arabidopsis thaliana*	TMV	CP	Maintains normal virion morphology in vascular tissues	Long-distance movement	Genetic evidence	The recessive Col-0 allele of *DSTM1* results in delayed TMV-U1 systemic movement	[69]
IP-L	*Nicotiana tabacum*, *Nicotiana benthamiana*	ToMV	CP	Interacts with CP to facilitate long-distance transport	Long-distance movement	Physical interaction; silencing	Silencing of *IP-L* delayed ToMV systemic movement	[70]

Footnote: For the “Interacting viral protein” column: Unknown indicates that no viral interaction partner has been experimentally characterized, and the corresponding molecular interaction remains unconfirmed.

**Table 2:** Summary of transcription factors, hormonal regulators, and PR proteins involved in tobamovirus resistance.

Category	Factor/Protein	Host Species	Induction Context/Functional Features	Reference
Transcription factor (TF)	WRKYb	*Capsicum annuum*	Directly binds the promoter of *CaPR10* and positively regulates innate immunity against TMV.	[117]
	CaBtf3 (basic TF 3)	*Capsicum annuum*	Transcriptionally activates HR-associated genes (*CaAlaAT1*) and PR genes (*CaPR1*, *CaPR10*); silencing compromises HR and increases viral accumulation.	[118]
	NAC1	*Nicotiana benthamiana*	Directly activates *ICS1* (SA biosynthesis gene) by promoter binding, boosting SA-mediated TMV resistance (feedback loop).	[119]
	MYB	*Nicotiana tabacum*	Facilitates SA signaling pathway activation to enhance TMV resistance upon induction by 9β-hydroxy-ageraphorone isolated from *Ageratina adenophora*	[120]
	MYB1	*Nicotiana tabacum*	Repression of MYB1 weakens HR triggered by viral infection.	[121]
	WRKY1–WRKY3	*Nicotiana tabacum*	Repression of these WRKYs significantly weakens HR triggered by viral infection.	[121]
	CaWRKYd	*Capsicum annuum*	Positive regulator of TMV resistance; silencing attenuates HR and increases CP accumulation. Induced by SA, MeJA, and ethephon.	[122]
	NbMYB4L	*Nicotiana benthamiana*	Activated by ethylene (ET) to trigger a MYB4L-dependent defense pathway against TMV.	[123]
Hormone/signaling	SA, MeJA, ethephon	*Capsicum annuum*	Upstream signals that induce expression of CaWRKYd, linking hormone perception to TF-mediated defense.	[122]
	Ethylene (ET)	*Nicotiana benthamiana*	Upregulates *NbMYB4L* to activate antiviral defense.	[123]
	SA pathway (ICS1)	*Nicotiana benthamiana*	Rate-limiting enzyme in SA biosynthesis; NAC1-mediated activation enhances TMV resistance.	[119]
PR protein	PR-1a, PR-O, PR-N, PR-2, PR2a, PR-3	*Nicotiana tabacum*	Accumulate during TMV-triggered HR; PR-O, PR-N, PR-2, PR2a exhibit β-1,3-endoglucanase activity; PR-3 has endochitinase activity.	[124,125,126]
	PR1, PR2, PR5	*Capsicum chinense*	Specifically induced during PMMoV-S incompatible interactions; earlier/stronger accumulation than in compatible interactions.	[127]
	CaGLP1	*Capsicum annuum*	Induced during TMV-P0 incompatibility; may strengthen cell walls (structural defense).	[128]
	CaPR-10	*Capsicum annuum*	Induced by TMV-P0; undergoes phosphorylation to gain RNase activity, directly degrading viral RNA and blocking proliferation.	[129]

**Table 3:** Key dominant and recessive tobamovirus resistance loci in solanaceous crops.

Crop	Gene/Locus	Resistance Type	Viral Target	Virus/Pathotype	Durability	Known Resistance-Breaking Isolates
Tobacco	*N*	NLR-based effector-triggered immunity	p50 helicase domain (indirect via NRIP1)	tobamovirus [137,155]	High	TMV-Ob [137]
Tobacco	*N’*	NLR-based effector-triggered immunity	CP	PMMoV, ToMV, paprika mild mottle virus [105], ToBRFV [155]	Moderate (poorly characterized under field conditions)	TMV-K98 [105]
Pepper	*L^1^*	NLR-based effector-triggered immunity	CP	P_0_ [138,139,140]	Low	P_1_, P_1,2_, P_1,2,3_, P_1,2,3,4_ [138,139,140], ToBRFV [112]
Pepper	*L^2^*	NLR-based effector-triggered immunity	CP	P_0_, P_1_ [138,139,140]	Moderate	P_1,2_, P_1,2,3_, P_1,2,3,4_ [138,139,140], ToBRFV [113]
Pepper	*L^3^*	NLR-based effector-triggered immunity	CP	P_0_, P_1_, P_1,2_ [138,139,140], ToBRFV [113]	Moderate	P_1,2,3_, P_1,2,3,4_ [138,139,140]
Pepper	*L* *^4^*	NLR-based effector-triggered immunity	CP	P_0_, P_1_, P_1,2_, P_1,2,3_ [138,139,140], ToBRFV [113]	High	P_1,2,3,4_ [138,139,140]
Pepper	*L^1a^*	NLR-based effector-triggered immunity	CP	P_0_, P_1_ (<26 °C) [114,115]	Low	P_1,2_, P_1,2,3_, P_1,2,3,4_ [114,115]
Tomato	*Tm-1*	Non-NLR replication-inhibitory resistance (tolerance)	130 kDa protein, 180 kDa protein	TMV [145], ToMV [147]	Moderate	ToMV1-2 [156], TMV-Ltal, TMV-L_11_Y237, TMV-CH2 [157], ToBRFV [142]
Tomato	*Tm-2*	NLR-based effector-triggered immunity (CC-NBS-LRR)	MP	TMV [158], ToMV [108]	Low	ToMV1-2 [156], ToMV-B7 [107], ToBRFV [143]
Tomato	*Tm-2^2^*	NLR-based effector-triggered immunity (CC-NBS-LRR)	MP	TMV [159], ToMV [108], dose-dependent partial resistance against ToBRFV [146]	High	ToMV-2(2) [160], ToBRFV [143]
Tomato	*TOM1*	Genome-edited resistance (validated in protoplasts)	Unknown	ToBRFV, ToMV [149]	Unvalidated in large commercial fields	None reported
Pepper	*TOM1*, *TOM3*	Experimentally induced susceptibility-gene knockdown	Unknown	TMV [150]	Unvalidated in large commercial fields	None reported
Tobacco	*TOM2A*	Genome-edited resistance (also naturally occurring in some cases)	Indirect (via TOM1; 126 kDa protein, 183 kDa protein)	TMV, ToMV [11,12]	Unvalidated in large commercial fields	None reported
Watermelon	*WPRb*	Naturally occurring recessive resistance	MP	CGMMV [151]	Unvalidated in large commercial fields	None reported
Tobacco	*eEF1A*	Experimentally induced susceptibility-gene knockdown	126 kDa protein, 183 kDa protein	TMV [152]	Unvalidated in large commercial fields	None reported
Tobacco	*ATPδ*	Experimentally induced susceptibility-gene knockdown	CP	CGMMV [153]	Unvalidated in large commercial fields	None reported
Tobacco	*KPILP*	Experimentally induced susceptibility-gene knockdown	Unknown	TMV [154]	Unvalidated in large commercial fields	None reported

**Table 4:** Summary of biological control agents and emerging chemical compounds with anti-tobamovirus activity.

Active Agent	Target Virus (Host)	Mechanism	Validation Level	Key Advantages	Major Limitations	Commercial/Regulatory Status
Biological control—Microbial metabolites and natural compounds						
Rhp-PSP (*Rhodopseudomonas palustris* JSC-3b) [254,255]	TMV (tobacco)	Endoribonuclease activity; R129 residue and homotrimeric assembly are essential for activity	Mechanistically established strategies; greenhouse validation	Mechanism defined; foliar sprayable	Protein stability in field unknown	Not registered
Extracellular proteins (*Pseudomonas fluorescens* CZ) [256]	TMV (tobacco)	Unknown	Greenhouse validation	Exhibits both preventive and curative antiviral activities.	Mechanism unclear; no field data	Not registered
Ribonuclease (*Bacillus cereus* ZH14) [257]	TMV (tobacco)	Unknown	Proof-of-concept laboratory studies	Thermostable; acid-resistant	No green-house/field validation	Not registered
Alkaline metalloproteinase (*Serratia marcescens* S3) [258]	TMV (tobacco)	Hydrolyzes TMV CP, disrupting virions	Greenhouse validation	Direct particle degradation	No field data; enzyme stability unknown	Not registered
*Acinetobacter* KNF2022 [259]	TMV (tobacco)	Unknown	Proof-of-concept laboratory studies	Root-associated strain; suppresses infection	Mechanism unclear	Not registered
Polypeptide extracts (*Penicillium chrysogenum*) [260]	TMV (tobacco)	Induces callose deposition at PD; inhibits cell-to-cell movement	Mechanistically established strategies; greenhouse validation	Mechanism defined	Crude extract; no field data	Not registered
Ningnanmycin (NNM) [242,261]	TMV (tobacco)	Activates MAPK/Ca^2+^ signaling; upregulates defense-related genes; systemic resistance	Commercial/regulatory status	Registered biopesticide; systemic	Moderate efficacy relative to several reported novel compounds	Registered in China
Methanolic Ammi visnaga seed extract (MKSE) [262]	ToMV (tobacco)	Direct viral inhibition; upregulates PR-2,induce callose at PD	Mechanistically established strategies; greenhouse validation	Dual action (direct inhibition + movement restriction); preventive, curative and virucidal activities all confirmed	Extract composition variable; no field data	Not registered
Isobavachalcone (IBC, from *Psoralea corylifolia* L.) [263]	TMV (tobacco)	Alleviates TMV-induced photosynthetic impairment; upregulates photosystem, carbon fixation, and energy metabolism proteins	Mechanistically established strategies; greenhouse validation	Mechanism defined	Indirect antiviral mechanism; no field validation	Not registered
Chitosan [264]	TMV (tobacco)	Activates endogenous proteases and RNases	Mechanistically established strategies; greenhouse validation	Mechanism defined	Efficacy decreases over time; no field validation	Not registered
Lentinan (LNT)/LNT + star polycation (SPc) nanocomplex [265]	TMV (tobacco)	Inactivates TMV particles; activates plant immunity	Mechanistically established strategies; field-tested approaches	Stable protective performance; improved leaf adhesion and uptake (SPc complex)	Poor leaf adhesion and uptake for LNT alone; complex formulation required	Not registered
Chitosan-coated lentinan-loaded calcium alginate hydrogel (CSL-gel) [266]	TMV (tobacco)	Modulates Ca^2+^ signaling; upregulates NbCML3 expression	Mechanistically established strategies; greenhouse validation	Long-term release; broad-spectrum activity confirmed against TMV, TRV, PVX, and TuMV	No field data; complex multi-component formulation	Not registered
Emerging chemical compounds						
Chlorine dioxide (ClO_2_) [267]	ToBRFV (tomato)	Oxidizes CP; breaks down genomic RNA; inhibits replication	Field-tested approaches	No phytotoxicity at 760 mg/L; improves agronomic parameters; degrades to harmless products	Excessively high doses trigger ROS overaccumulation and leaf phytotoxicity	Not registered
Seco-pregnane C21 steroid derivatives [268]	TMV (tobacco)	Unknown	Proof-of-concept laboratory studies(antiviral activity in local-lesion assays)	Several derivatives (5g, 5j, 5l) show higher anti-TMV activity than ningnanmycin	Mechanism unclear; no field data	Not registered
Chlorin e6 (Ce6) + LED light [269]	TMV (tobacco)	Light-activated Ce6 generates ROS that selectively cleave the G4-forming RNA sequence in the TMV genome	Mechanistically established strategies; greenhouse validation	Target-specific (G4 sequence)	Light-dependent;	Not registered
SN11 (cytidine peptide derivative) [270]	TMV (tobacco)	Induces differential expression of genes involved in ribosome biogenesis/function, plant hormone signal transduction, plant–pathogen interactions, and chromatin (RNA-seq defined)	Mechanistically established strategies; greenhouse validation	Triple modes: protective, virucidal, and curative activities	No field data	Not registered
Ferulic acid dimer A6 [271]	TMV (tobacco)	Binds TMV CP; disrupts viral particle integrity	Mechanistically established strategies; proof-of-concept laboratory studies (antiviral activity in local-lesion assays)	Direct CP binding	No greenhouse/field validation	Not registered

## Data Availability

No new data were created or analyzed in this study. Data sharing is not applicable to this article.
